# The value of genome-wide analysis in craniosynostosis

**DOI:** 10.3389/fgene.2023.1322462

**Published:** 2024-01-22

**Authors:** Alexandra Topa, Anna Rohlin, André Fehr, Lovisa Lovmar, Göran Stenman, Peter Tarnow, Giovanni Maltese, Madiha Bhatti-Søfteland, Lars Kölby

**Affiliations:** ^1^ Department of Laboratory Medicine, University of Gothenburg, Sahlgrenska Academy, Gothenburg, Sweden; ^2^ Department of Clinical Genetics and Genomics, Sahlgrenska University Hospital, Gothenburg, Sweden; ^3^ Department of Pathology, Sahlgrenska University Hospital, Gothenburg, Sweden; ^4^ Department of Plastic Surgery, University of Gothenburg, Sahlgrenska Academy, Gothenburg, Sweden

**Keywords:** genetic, gene, suture, syndrome, skull, craniofacial, synostosis

## Abstract

**Background:** This study assessed the diagnostic yield of high-throughput sequencing methods in a cohort of craniosynostosis (CS) patients not presenting causal variants identified through previous targeted analysis.

**Methods:** Whole-genome or whole-exome sequencing (WGS/WES) was performed in a cohort of 59 patients (from 57 families) assessed by retrospective phenotyping as having syndromic or nonsyndromic CS.

**Results:** A syndromic form was identified in 51% of the unrelated cases. A genetic cause was identified in 38% of syndromic cases, with novel variants detected in *FGFR2* (a rare Alu insertion), *TWIST1, TCF12, KIAA0586, HDAC9, FOXP1*, and *NSD2*. Additionally, we report two patients with rare recurrent variants in *KAT6A* and *YY1* as well as two patients with structural genomic aberrations: one with a 22q13 duplication and one with a complex rearrangement involving chromosome 2 (2p25 duplication including *SOX11* and deletion of 2q22). Moreover, we identified potentially relevant variants in 87% of the remaining families with no previously detected causal variants, including novel variants in *ADAMTSL4, ASH1L, ATRX, C2CD3, CHD5, ERF, H4C5, IFT122, IFT140, KDM6B, KMT2D, LTBP1, MAP3K7, NOTCH2, NSD1, SOS1, SPRY1, POLR2A, PRRX1, RECQL4, TAB2, TAOK1, TET3, TGFBR1, TCF20*, and *ZBTB20*.

**Conclusion:** These results confirm WGS/WES as a powerful diagnostic tool capable of either targeted *in silico* or broad genomic analysis depending on phenotypic presentation (e.g., classical or unusual forms of syndromic CS).

## 1 Introduction

Craniosynostosis (CS) represents the premature closure of the skull sutures and occurs in ∼1 in 1,300 live births in the Swedish population (Tarnow et al., 2022). CS occurrence in the absence of other developmental anomalies and/or psychomotor delay is classified as nonsyndromic CS (NCS) and represents the most common presentation observed in 70%–75% of all patients with CS (Lattanzi et al., 2017; Armand et al., 2019).

Another classification is based on the type of suture(s) involved (sagittal, coronal, metopic, lambdoid) and can be restricted to a single suture or a multisuture form, such as pansynostosis, where all sutures are affected. Isolated sagittal synostosis is the most frequent form of CS, occurring in 45%–58% of all NCS cases (Lajeunie et al., 1996). Multisuture CS, specifically bicoronal synostosis, is frequently observed in syndromic forms of CS (SCS) (Twigg and Wilkie, 2015).

The etiology of CS is largely unknown. The interplay between genetic and environmental factors appears to play a role, especially in isolated NCS (Timberlake and Persing, 2018). However, genetic factors have a determinant role in the etiology of SCS, and there are several known core genes (*FGFR1, FGFR2, FGFR3, TWIST1*) associated with classical CS syndromes, such as Crouzon, Saethre–Chotzen, Pfeiffer, Muenke, and Apert (Muenke et al., 1994; Reardon et al., 1994; Wilkie et al., 1995; Bellus et al., 1996; Howard et al., 1997). Additional genes have been identified in other less frequently observed syndromes where CS is a main symptom, including craniofrontonasal (*EFNB1*), Antley–Bixler (*POR*), Carpenter types 1 (*RAB23*) and 2 (*MEGF8*), and CS and dental anomalies (*IL11RA*) (Flück et al., 2004; Twigg et al., 2004; Jenkins et al., 2007; Nieminen et al., 2011; Twigg et al., 2012). The development of whole-exome and whole-genome sequencing (WES/WGS) technologies has facilitated the discovery of new genes associated with CS syndromes, such as *ERF* (CS type 4), *TCF12* (CS type 3), and *P4HB* and *SEC24D* (Cole–Carpenter types 1 and 2), as well as genes involved in syndromes where CS is a variable feature [e.g., Weiss–Kruszka (ZNF462) and Say–Barber–Biesecker–Young–Simpson (*KAT6B*) syndromes] (Clayton-Smith et al., 2011; Sharma et al., 2013; Twigg et al., 2013; Garbes et al., 2015; Rauch et al., 2015; Weiss et al., 2017). Moreover, mutations in *SMAD6* have been detected by WES in subsets of patients with midline NCS (Timberlake et al., 2016). Notably, up to 14% of CS cases are caused by chromosomal aberrations (Wilkie et al., 2010). Low-penetrant mutations in CS core genes (*FGFR2, FGFR3, TWIST1*) have been detected in NCS presentations, underlining the existence of a phenotypic continuum between NSC and SCS (Heuzé et al., 2014).

Phenotypic presentation plays an important role in the genetic testing strategy for CS patients. Targeted screening covering CS core genes (*FGFR2, FGFR3, TWIST1, FGFR1, EFNB1, TCF12, ERF*) has a high diagnostic yield of up to 90% in SCS patients with the most recognizable classical phenotypes. In particular, bicoronal synostosis is an indicator of SCS (Wilkie et al., 2017). However, considering the significant variable expressivity and phenotypic heterogeneity of CS syndromes, recent studies demonstrate the advantage of next-generation sequencing (NGS) using gene panels with less-frequently mutated CS core genes, such as *IL11RA, POR, MSX2,* and *CDC45*. Furthermore, WES and WGS enable targeted *in silico* analysis of a custom-designed CS-related gene panel and performance of a broader exome/genome analysis in cases returning negative results. This is a cost-effective strategy that increases the diagnostic yield and can be adapted to the phenotypic presentation (Miller et al., 2017; Tønne et al., 2020; Hyder et al., 2021; Bukowska-Olech et al., 2022; Tønne et al., 2022).

The aim of the present study was to use WES and WGS to search for rare genetic variants that can explain CS within a cohort of clinically well-characterized SCS and NCS patients that had previously undergone targeted mutation screening (see 2.2 Methods) with negative results.

## 2 Materials and methods

### 2.1 Patient data

A total of 59 patients with CS from 57 families (one family comprises the mother and her two sons with similar phenotype) was included in the study. The following inclusion criteria were applied: patients with mostly coronal or multiple suture synostosis with either syndromic or nonsyndromic presentation, and without a detected pathogenic or likely pathogenic variant at previous targeted testing. Thirty-seven patients up to 2016 were retrieved from the Gothenburg Craniofacial Registry at the Sahlgrenska University Hospital, and the remaining 22 patients were retrieved from the clinical laboratory records for patients with negative outcomes following mutation screening between 2016 and 2020. Patient phenotypes were assessed retroactively by corroborating the medical records with registered photos and three-dimensional computed tomography reconstructions. A patient was considered to have a syndromic form of CS if one or several of the following features were present: 1) craniofacial changes involving the eyes (e.g., proptosis, hypertelorism), maxilla (e.g., midface retrusion with relative prognathism, micrognathia), nasal pyramid (e.g., short, small, beaked), and ears (e.g., low-set, dysplastic); 2) neurodevelopmental abnormalities (e.g., motor and/or speech delay, intellectual disability, seizures); and 3) other associated malformations (e.g., cleft palate, heart defect).

### 2.2 Methods

#### 2.2.1 Genetic screening prior to study inclusion

Thirty-seven patients were initially analyzed using a custom-designed NGS 63 CS-gene panel (Topa et al., 2020; Topa et al., 2022). Twenty-one patients were analyzed in a clinical setting using either an *in silico* CS-gene panel on a WES (6 patients)/WGS platform (15 patients) or a targeted NGS 12 CS-gene panel (1 patient) using the HaloPlex system (Agilent Technologies, Santa Clara, CA, United states) (Supplementary Methods S1). Complementary multiplex ligation-dependent probe amplification covering 10 genes (*TWIST1, FGFR1, FGFR2, FGFR3, MSX2, ALX1, ALX3, ALX4, EFNB1, RUNX2*) was performed for all 21 clinically screened cases.

#### 2.2.2 Genetic screening for the present study

Genomic DNA was extracted from blood samples. Parental samples were not systematically collected but obtained *a posteriori* in selected cases with phenotypically relevant variants for subsequent segregation analysis. The majority of patients were analyzed as singletons, except for three cases, where parental and sibling samples were available for Trio, Quatro, and Duo analysis, respectively. Overall, data from 52 WGS and five WES analyses of index family cases were processed for this study (Supplementary Methods S1).

All 57 index family cases were initially screened using an *in silico* panel that included 133 genes using Alissa Interpret (Agilent Technologies) for the variant-filtration pipeline (Supplementary Methods S1). The genes were selected according to their association with a CS phenotype and using PubMed and OMIM databases.

WGS and WES analyses were performed by filtration of variants from vc files and those containing copy number variations (CNVs) using the following software programs: Moon (Invitae Corp., San Francisco, CA, United States) comprising human phenotype ontology (HPO)-term-driven analysis; and Alissa Interpret (Agilent Technologies) using a classic variant-filtration pipeline, including primarily population frequency, inheritance pattern, molecular aspects, and CS-related HPO terms. Assessment of CNVs was performed on WGS data using the described variant-interpretation software and the Integrative Genomics Viewer (https://software.broadinstitute.org/software/igv/). Clinically relevant sequence variants and CNVs not fulfilling technical quality thresholds were confirmed by Sanger sequencing and multiplex ligation-dependent probe amplification or SNP array. mRNA/cDNA analysis was performed for novel variants with predicted splice effects.

Variant interpretation and classification were performed manually based on criteria, such as minimal allele/genotype frequency in gnomAD (https://gnomad.broadinstitute.org/), genotype–phenotype correlation, location at protein level, and evolutionary conservation. Additionally, computational data were evaluated using Alamut Visual Plus software (Sophia Genetics, Lausanne, Switzerland) with integrated *in silico* prediction tools, such as SIFT (https://bio.tools/sift), polyphen2 (http://genetics.bwh.harvard.edu/pph2/), and MutationTaster (https://www.mutationtaster.org/). The results were also compared with variant databases, such as HGMD professional (https://my.qiagendigitalinsights.com/bbp/) and ClinVar (https://www.ncbi.nlm.nih.gov/clinvar/), in combination with in-house information. Variant classification was performed in accordance with American College of Medical Genetics and Genomics guidelines (Richards et al., 2015).

## 3 Results

### 3.1 Clinical findings

The gender proportion was 1:1.2 (27 males and 32 females). A syndromic form of CS was recognized in 51% of families. The distribution of the sutural pattern in the SCS and NCS groups is depicted in Supplementary Figure S1. In the multiple synostosis group (20 cases, including 16 SCS), the involvement of different sutures was as follows: coronal, 16 cases; sagittal, 13 cases; lambdoid, seven cases (5 combined with coronal); metopic, four cases (3 combined with coronal and 1 with sagittal); frontosphenoidal, two cases (combined with coronal); and pansynostosis, one case. Combined coronal and sagittal involvement were observed in 10 of 20 cases and predominantly in SCS. Mercedes synostosis (sagittal and bilateral lambdoid) was noted in two SCS cases. The phenotypic details, including the sutural pattern for each patient, are shown in Supplementary Table S1.

### 3.2 Genetic findings

Molecular outcomes according to phenotypic presentation and suture pattern are depicted in Supplementary Figures S2, S3. We detected causal (pathogenic or likely pathogenic according to the ACMG criteria) variants in 19% of the families and 38% of unrelated patients with SCS (Table 1). Seven causal and novel variants were detected in *FGFR2, TWIST1*, *TCF12, KIAA0586, HDAC9, FOXP1*, and *NSD2*, and a *de novo* deletion in *HDAC9* (P2605_132) detected using the *in silico* panel on WGS data was confirmed by multiplex ligation-dependent probe amplification. In this case, the breakpoints were located upstream of *TWIST1,* and the deletion included *HDAC9*, *PRPS1L1*, and *SNX13* (Supplementary Figure S4), none of which had been clearly associated with disease in humans. The typical Apert phenotype in one patient (P_3) prompted a detailed examination of *FGFR2*, which revealed insertion of an Alu element 18-bp upstream of the boundary between intron 7 and exon 8 (NM_000141.5). This was subsequently confirmed by targeted NGS analysis at an external lab. Additionally, we identified the novel splice variant in *TCF12* in our clinical screening and classified it as a variant of uncertain significance. The variant was subsequently upgraded to likely pathogenic after complementary parental segregation and mRNA analyses. cDNA sequencing revealed introduction of a cryptic acceptor splice site (TTTCTAG) at position −8 of intron 18, leading to a frameshift and an early stop codon. Furthermore, we detected larger *de novo* structural genomic rearrangements in two patients (P_2 and P2605_175) (Table 1). For patient P2605_175, we were unable to confirm the breakpoints in the complex rearrangement involving chromosome 2 by SNP array. Supplementary Table S2 outlines additional findings in CS-relevant genes with possible modulator effects, detected in patients with causal variants.

**TABLE 1:** Causal variants detected by WGS in patients with syndromic SCS.

Patient no. (gender)	Suture pattern	Phenotype (clinically suspected diagnosis)	Analyses prior to inclusion in the study (on both clinical and research bases)	Gene(transcript)/Chromosomal aberration	Variant annotation - cDNA, protein level/genomic position for CNVs (size)	Variant classification according to ACMG criteria (novelty, zygosity, inheritance, molecular aspects^a^)	Detection by screening method			Associated phenotype/disorder (OMIM, PubMed - PMID)
							*In silico* panel on WGS/WES (133 genes)	HPO-term analysis with Moon/Alissa software	CNV analysis (Alissa + IGV)	
**P2603_144** ^b^ ^,^ ^c^ (M)	Bicoronal	SCS (Noonan, Loeys-Dietz, Baraitser-Winter, BPES, Ohdo-like)	SNP-array, targeted NGS panels (Noonan, Marfan, CS-63 genes), WES Trio	***KIAA0586*** *(TALPID3)* NM_001329943.2	c.392delG, p. (Arg131Lysfs*4)	Pathogenic (compound het, maternal) 0.6207% (773 het) and 0.0016059% (2 hom) in gnomAD, truncating frameshift, reported as pathogenic by several sources in the literature and databases, such as ClinVar (VCV000204593.49) and HGMD (CD155358)	—	+ (only Moon)	—	Joubert syndrome 23, Autosomal recessive (#616490)
					c.887A>G, p. (Tyr296Cys)	Likely pathogenic^d^ *(novel)* (compound het, paternal) 0.0064241% (8) in gnomAD, missense, highly conserved, large physicochemical difference, in *trans* with pathogenic variant				
**P2605_115(M)**	Sagittal + lambdoid (Mercedes synostosis)	SCS (Catel-Manzke-like face)	Targeted Sanger (*FGFR, TWIST, POR*) and NGS panel (63 genes)	***YY1*** NM_003403.4	c.1057T>C, p. (Phe353Leu)	Likely pathogenic^e^ (het, expected *de novo*, absent in the mother, father unavailable) Absent from gnomAD, missense in protein domain, highly conserved, small physicochemical difference, 4/4 damaging, possible splice effect, recently reported as likely pathogenic in ClinVar (VCV002413123.4)	+	+	—	Gabriele-de Vries syndrome (#617557)
**P2605_105^c^ (F)**	Unicoronal right	SCS (Saethre-Chotzen)	Targeted Sanger (*FGFR, TWIST, POR*) and NGS-panel (63 genes)	***TWIST*** *1*	Whole-gene deletion (including *FERD3L*) arr [GRCh37] 7p21.1 (19066802_19662813)x1 (596 kb)^f^	Pathogenic (het, *de novo*)	+	+	+	Saethre-Chotzen syndrome (#101400)
**P2605_102 (M)**	Bicoronal + metopic + sagittal (?)	SCS (Shprintzen-Goldberg-like, atypical)	Targeted NGS panel (63 genes)	***KAT6A*** NM_006766.5	c.3055C>T, p. (Arg1019*)	Pathogenic^e^ (het, expected *de novo*) Absent in gnomAD, truncating frameshift, possible splice effect, reported as *de novo* pathogenic in ClinVar (VCV000489088.3) and HGMD (CM194936)	+	+	—	Arboleda-Tham syndrome (#616268)
**P2605_132^c^** ^**,**^ **^g^ (F)**	Unicoronal right	SCS (Saetre-Chotzen-like, Branchiootic – like)	Targeted Sanger (*FGFR1, FGFR2, FGFR3, TWIST1)* and NGS panel (63 genes)	***HDAC9***	Whole-gene deletion (including *PRPS1L1, SNX13*) arr [GRCh37] 7p21.1 (17930686_19059254)x1 (1,128 kb)^f^	Likely pathogenic *(novel)* (het, *de novo*) Absent from gnomAD	+	—	+	Branchiootic-syndrome (BOS)-like phenotype (PMID: 35710300)
**P2605_175^c^ (F)**	Unicoronal right	SCS (Saethre-Chotzen-like)	Targeted Sanger *(FGFRs, TWIST1, POR),* NGS panel (63 genes)	**Complex structural rearrangement: Dup2p25 (1.5** ** ** **Mb) + Del2q22 (3,4** ** ** **Mb)** ^h^	arr [GRCh37] 2p25.2p25.1 (5689487_7379378)x3 (1,7 Mbp) arr [GRCh37] 2q22.1 (138458639_141918297)x1 (3,4 Mbp)	Likely pathogenic *(novel)* (het, *de novo*)	—	—	+	Candidate genes for the phenotype: Dup2p25.2:*SOX11* (#615866, AD) Del2q22.1: *HNMT* (#616739,AR, no other variant detected)+*LRP1B* (*608766)*+NXPH2* (*604635)
**P_1^c^ (F)**	Bicoronal	SCS	*In silico* panel WGS (29 genes)+MLPA	***TCF12*** NM_207036.1	c.1746-8T>G, p. (Ser583Phefs*5)	Likely pathogenic *(novel)* (het, *de novo*) Absent from gnomAD, high probability of splice-effect confirmed by mRNA/cDNA analysis	+	+	—	Craniosynostosis 3 (#615314)
**P_2^b^** ^**,**^ **^c^ (F)**	Sagittal	SCS	HaloPlex NGS-panel (12 genes)+MLPA	**Duplication 22q13.1-q13.2**	arr [GRCh37] 22q13.1q13.2 (40545592_42096995)x3 (1.55 Mb)^f^	Likely pathogenic *(novel)* (het, *de novo*)	—	+ (only Moon)	+	Chromosome 22q13 duplication syndrome (#615538) Candidate gene for the phenotype: *EP300*
**P_3^c^ (F)**	Bicoronal + lambdoid (?)	SCS (Apert, typical)	*In silico* panel on WGS (29 genes) + MLPA	***FGFR2*** NM_000141.5	c.940-19_c.940-18insAlu	Likely pathogenic *(novel)* (het, *de novo*) Absent in gnomAD, Alu-insertion of 18 basepairs upstream from the intron 7 – exon 8 boundary, possibly affecting splicing, previously reported Alu-insertions impacted splicing	—	—	+	Apert syndrome (#101200)
**P_4 (M)^g^**	Sagittal	SCS	SNP-array, Fragile X, *in silico* panel WGS (29 genes)+MLPA	***FOXP1*** NM_001349338.3	c.910G>T, p. (Gly304*)	Pathogenic *(novel)* (het, *de novo*) Absent from gnomAD, truncating, similar LoF variants reported as pathogenic	—	+ (only Moon)	—	Intellectual developmental disorder with language impairment with or without autistic features (# 613670)
**P_5 (F)^g^**	Sagittal	SCS (Crouzon, atypical)	*In silico* panel WGS (29 genes)+MLPA	***NSD2*** *(WHSC1)* NM_133330.3	Ex 1–8 deletion arr [GRCh37] 4p16.3 (1877218_1934173)x1 (57 kb)^i^	Likely pathogenic *(novel)* (het, *de novo*)	-	+ (only Moon)	+	Rauch-Steindl syndrome (# 619695)

^a^ Genotype frequency in control population (gnomAD), effect at protein level, location in protein domain, nucleotide/amino acid evolutionary conservation, physiochemical difference between amino acids, no/4 – number of *in silico* prediction programs assessing the variant as damaging/tolerated per total number of programs (4): SIFT, MutationTaster, PolyPhen-2: HumDiv and HumVar.

^b^ Parental samples were available for WGS, trio analysis.

^c^ Secondary findings with potentially modulator effect (Supplementary Table S2).

^d^ Considerations for the variant classification: compound heterozygosity with a pathogenic variant and the genotype–phenotype correlation.

^e^ Considerations for the variant classification: expected *de novo* occurrence, previous report with *de novo* occurrence, and the genotype–phenotype correlation.

^f^ Structural variant confirmed by MLPA., the breakpoints were manually curated, the array annotation is suggested by the variant interpretation program.

^h^ Confirmed by microarray (Affymetrix CytoScan HD). Parental samples were analyzed using both microarray and karyotyping in order to exclude a balanced structural rearrangement.

^g^ Additional discarded variants due to their unlikelihood to contribute to the phenotype (Supplementary Table S5).

^i^ The breakpoints were manually curated. Parental samples were analyzed with microarray (no MLPA, kit available).

(−) variant not detected by method; (+) variant detected by method; BOS, Branchiootic syndrome; BPES, Blepharophimosis, epicanthus inversus, and ptosis syndrome; ClinVar, Clinical Genome Resource (database of variants associated with human disease); F, female; gnomAD, The Genome Aggregation Database; het, heterozygous; HGMD, Human Gene Mutation Database; LoF, loss-of-function; M, male; MLPA, multiplex ligation-dependent probe amplification; VUS, variant of uncertain significance.

Novel and possibly relevant variants were detected in 87% of families in which no causal variants were observed (Supplementary Tables S3, S4). When parental samples were available, segregation analysis was performed in cases harboring very low frequency variants in control populations and predicting damaging effects. Thirty-four novel and possibly relevant variants were detected in *ADAMTSL4, ASH1L, ATRX, C2CD3, CHD5(2), DMP1, ERF, H4C5, IFT122 (2), IFT140, KDM6B, KMT2D, LTBP1(2), MAP3K7, NOTCH2, NSD1(2), SOS1, SPRY1, POLR2A, PRRX1, RECQL4, TAB2, TAOK1(3)*, *TET3(2), TGFBR1, TCF20*, and *ZBTB20*. New causal and possibly relevant variants were detected in 23 of 37 patients previously screened using the targeted 63-gene NGS panel. Eight variants that had been observed but not reported at previous targeted NGS analysis were confirmed with WGS and reassessed as possibly relevant (Supplementary Tables S3, S4). Additional variants, including novel ones considered unlikely to contribute to the phenotype and/or discarded after segregation analysis, are listed in Supplementary Table S5.

A total of 115 genetic variants were detected using a combination of the *in silico* panel along with HPO-driven and CNV analyses. The majority of variants (89%) were detected by HPO-driven filtration, of which 50% were identified exclusively by one software program (Moon). The *in silico* panel detected 41% of the variants, of which 8% were identified exclusively by this filtration method. Six of the causal variants (5.2%) were detected by CNV analysis combined with the *in silico* panel and HPO filtration in four cases (Table 1). Overall, causal and potentially significant variants were identified in 89% of the unrelated patients. The contribution of WGS/WES to the diagnostic yield (causal variants) was 16% for patients initially analyzed using the targeted 63-gene NGS panel and 25% for patients initially analyzed using the *in silico* clinical panel.

## 4 Discussion

In this study we applied WES or WGS analyses of a cohort of patients with SCS and NCS and that previously tested negative in a targeted mutation screening program (Topa et al., 2020; Topa et al., 2022). The results revealed a diagnostic yield of 38% in patients with SCS and possibly relevant variants in 87% of the remaining patients (NCS included).

### 4.1 Main results

We report causal and likely causal variants (whereof eight novel) in the following genes (Table 1): 1) CS core genes *FGFR2, TWIST1,* and *TCF12*; 2) *HDAC9*, a gene situated in a candidate region with regulatory elements for *TWIST1*; 3) genes occasionally associated with CS (e.g., *FOXP1*, *NSD2*, and *YY1*); and 4) *KIAA0586*, which was not previously associated with CS. Furthermore, we report two *de novo* chromosomal aberrations (a microduplication of 22q13.1-q13.2 and a complex rearrangement of chromosome 2) and an additional case of a recurrent pathogenic variant in *KAT6A*.

The previously reported pathogenic variant in *KIAA0586* (centrosomal protein homolog to chicken TALPID3) is the most frequently identified variant in Joubert syndrome type 23. This variant has been observed in a homozygous state in two individuals in the control population, suggesting a hypomorphic allele with pathogenic effect only in a compound heterozygous state with more severe variants (Pauli et al., 2019). The phenotype of the CS patient harboring this variant was not suggestive of Joubert syndrome, although some of the features, such as gross motor delay with impaired balance and coordination, were indicative of cerebellar abnormalities. To the best of our knowledge, this is the first case of CS among *KIAA0586*-related disorders, which are hybrid ciliopathies with a broad phenotypic spectrum ranging from severe skeletal abnormalities to Joubert syndrome. As such, patients may present a clinical picture that overlaps with these entities (Alby et al., 2015; Malicdan et al., 2015).

Recurrent *de novo* occurrence of the same pathogenic variant in *KAT6A* was reported by Kennedy et al. (2019) in two patients, one of which presented with sagittal CS. Microcephaly and CS are recurrent features in Arboleda−Tham syndrome, with several cases of midline synostosis and pansynostosis described in association with truncating variants in *KAT6A* (Tham et al., 2015; Kennedy et al., 2019; Timberlake et al., 2019; Marji et al., 2021; Korakavi et al., 2022). Interestingly, Clarke et al. (2018) reported two rare missense variants in *KAT6A* in three cases of isolated NCS (two metopic and one coronal).

There is only one report of CS in Gabriele-de-Vries syndrome (Gabriele et al., 2017), with that describing a likely pathogenic *de novo* missense variant in *YY1* encoding a transcription factor [c.1015A>C, p. (Lys339Gln)]. However, facial asymmetry and a broad head have been described in other patients, suggesting that mild CS may be underdiagnosed. Furthermore, the variant in our patient [c.1057T>C, p. (Phe353Leu)] is expected to have occurred sporadically and is located in the same zinc-finger protein domain as the variant reported by Gabriele et al. (2017), thus supporting its potential pathogenic role.

To the best of our knowledge, this study includes the second reported case of CS associated with a *de novo* truncating variant in *FOXP1*. Urreizti et al. (2018) reported a patient with metopic synostosis and an Opitz C-trigonocephaly-like phenotype. The sagittal synostosis in our patient may have occurred independent of the *FOXP1* variant, explaining only the neurodevelopmental symptoms. However, recent transcriptome studies show that neural crest-derived cells from the sagittal suture of human embryonic calvaria are highly enriched for the FOXP1/2 transcriptional network (He et al., 2021). Moreover, a truncating variant in *FOXP2* inherited from an affected parent was recently reported in a patient with sagittal SCS (Tønne et al., 2022). This further supports the involvement of *FOXP1/2* in midline synostosis.

The deletion identified in *NSD2* is to the best of our knowledge the first limited to exons 1 through 8. Larger structural variants (SVs) involving *NSD2* are associated with Wolf–Hirschhorn 4p16.3 deletion syndrome, and loss-of-function (LoF) and missense variants in *NSD2* were recently associated with the developmental disorder Rauch–Steindl syndrome (Zanoni et al., 2021). In that study, CS was diagnosed in one of the patients with a *de novo* missense mutation in *NSD2*, but the authors attributed this to the co-occurrence of a second *de novo* missense mutation in *AGO2*, which is associated with skull deformities, such as scaphocephaly. However, the microcephaly, dolichocephaly, and craniofacial asymmetry described in Rauch–Steindl syndrome suggest that CS is underdiagnosed. The present findings support that CS may occasionally be part of Rauch–Steindl syndrome.

We report the fourth case of CS and deletion involving *HDAC9* without disrupting the *TWIST1*-coding region. Recently, Hirsch et al. (2022) showed that SVs involving *HDAC9* disrupt *TWIST1*-regulatory elements within *HDAC9* in patients with CS. Our patient had coronal synostosis that was also described in two of the previously reported patients with deletion and one with a translocation breakpoint disrupting the *HDAC9–TWIST1* locus (Hirsch et al., 2022). Interestingly, the craniofacial features of our patient during infancy were not suggestive of Sathre–Chotzen syndrome. No information about possible syndactyly was available, and the presence of dysplastic helices, hearing loss, and a branchial cyst suggested branchiootic syndrome.

CS has not been documented in previous cases of 22q13.1-q13.2 duplications, although prominent forehead with brachycephaly are recurrent features (Samanich et al., 2012; Rahikkala et al., 2013). Notably, *de novo* mutations in *EP300* (included in the 22q13 duplication) are suggested to result in gain-of-function and are associated with a phenotype distinct from Rubinstein–Taybi syndrome caused by LoF variants (Menke et al., 2018). The facial appearance with prominent forehead and short up-slanting palpebral fissures are reminiscent of features observed in association with 22q13.1-q13.2 duplications, suggesting *EP300* as a candidate gene for the phenotype. However, the sagittal synostosis in our patient could be related to the paternally inherited *MSX2* variant with incomplete penetrance.

Another interesting finding was the *de novo* complex genomic rearrangement, including a 2p25 duplication and 2q22 deletion in a patient with a Saethre–Chotzen-like phenotype. The duplication involves *SOX11*, in which a *de novo* missense variant was reported in a patient with lambdoid synostosis and Coffin–Siris features (Timberlake et al., 2019). We could not determine a clear correlation between the phenotype and the genes included in the 2q22 deletion (containing the known protein-coding genes *HNMT, LRP1B*, and *NXPH2*). Only *HNMT* is disease-associated, specifically with a recessive type of intellectual disability (OMIM # 616739). Both *LRP1B* and *NXPH2* are dosage-sensitive and likely intolerant to haploinsufficiency.

Alu insertions represent a very rare mutational mechanism in Apert syndrome. To the best of our knowledge, there are only three previously reported cases caused by similar Alu insertions (Oldridge et al., 1999; Bochukova et al., 2009). We recommend undertaking specific searches for this type of mutation in patients with typical Apert syndrome and without coding variants in *FGFR2*.

Overall, we observed enrichment of causal variants in developmental genes, including those encoding transcription factors and chromatin modifiers (e.g., *FOXP1, KAT6A, NSD2, YY1*) associated with several disorders with pleiotropic phenotypes. CS is a variable feature in these pathologies, which may depend on the widespread downstream transcriptional and epigenetic effects of these genes (Zollino et al., 2017).

### 4.2 Additional results

#### 4.2.1 Additional relevant variants *in SCS*

The variant in *ATRX* (Supplementary Table S3) may be related to the Carpenter-like phenotype observed in patient P2605_136. A recent report identified a missense variant (NM_000489: c.6511A>G p. Met2171Val) in the C-terminal helicase domain of *ATRX* in two brothers with a Carpenter-like phenotype (patient photos not provided) accompanied by coronal and sagittal synostosis (Sáenz et al., 2021). However, the same variant was reported in a patient with ATR-X syndrome in the absence of noticeable skeletal abnormalities (Wada et al., 2013). The missense variant in our patient (c.690T>G, p. (Ile230Met)) is located in a zinc-finger protein domain in which neighboring mutations have previously been associated with ATR-X syndrome (Gibbons et al., 2008; Arvio and Lähdetie, 2021).

The novel *TET3* (Supplementary Table S3) variants in the patient with multisuture synostosis are of particular interest for CS. This case and previous reports of patients with *TET3*-related craniofacial involvement including brachycephaly and asymmetric skull shapes point out towards a possible role of *TET3*-variants in the dysregulation of gene expression during suture development (Beck et al., 2020; Seyama et al., 2022).

Additionally, we found novel variants in *POLR2A* and *PRRX1* (Supplementary Table S3) genes that have recently been associated with metopic and lambdoid synostosis, respectively (Tønne et al., 2022; Timberlake et al., 2023). Interestingly, previous studies demonstrated that PRRX1 is expressed in calvarial sutural mesenchymal stem cells (Wilk et al., 2017).

Notably, we observed two patients with pathogenic and likely pathogenic variants in *GSK* and *ABCC8* (Supplementary Table S3), respectively, in which mutations lead to familial hyperinsulinemic hypoglycemia. The *GSK* variant explained the recurrent hypoglycemia in the patient with bicoronal synostosis. Because activation of the IGF1-signaling pathway is involved in CS pathogenesis (Al-Rekabi et al., 2016; Gustafson et al., 2019), we cannot rule out a possible role for the *GSK* and *ABCC8* variants in CS occurrence in these patients.

#### 4.2.2 Additional relevant variants *in NCS*

We found an inherited novel variant in *ERF* in a patient with unicoronal synostosis, and two variants in *SPRY1* in two patients with multiple synostosis and involvement of the sagittal suture (Supplementary Table S4). The variants were inherited from an *a priori* unaffected parent. A truncating variant in *SPRY1* was reported by Timberlake et al. (2016) in a woman with mild cranial dysmorphism and her two children with sagittal synostosis. Tooze et al. (2023) also reported a case of syndromic sagittal synostosis with complete LoF of SPRY1 due to bi-allelic inheritance of a truncating variant from healthy parents. These findings suggest that heterozygous *SPRY1* variants may play a modifier role in the occurrence of sagittal NCS, whereas bi-allelic LoF variants lead to a more severe phenotype with SCS.

Additionally, we observed enrichment of possibly relevant variants in genes involved in the TGF-β pathway (*ADAMTSL4, LTBP1*, *LTBP4* and *TGFBR1* - Supplementary Table S4) with a role in the morphogenesis of the skull sutures (Rawlins and Opperman, 2008). Furthermore, CS was recently reported in patients with bi-allelic mutations in *ADAMTSL4* and *LTBP1* (Pottie et al., 2021; Gustafson et al., 2022). Also, we observed enrichment of heterozygous carriers of variants in CS-related autosomal recessive genes from the ciliopathy spectrum (e.g., *IFT122*, *IFT140*, and *WDR19* - Supplementary Table S4). These findings, together with results from previous studies showing enrichment of damaging variants in genes associated with SCS in patients with NCS, suggest an oligogenic involvement with incomplete penetrance in the occurrence of single-suture NCS (Clarke et al., 2018; Gustafson et al., 2019; Tiberio et al., 2021).

## 5 Limitations

This study has limitations in its retrospective nature, reduced number of patients and partly limited access to phenotypic details. An additional limitation is the lack of parental samples, which complicated the interpretation of variants of uncertain significance. Also, the cost of high-throughput sequencing methods limited the access to WGS/WES Trio in those cases where parental samples were obtained *a posteriori* for targeted segregation analysis of detected variants in singleton WGS/WES analyses. Another limitation resides in the capacity of the bioinformatic pipeline to detect CNVs/SVs from genomic data, which results in the possibility that certain smaller SVs, indels, or intragenic deletions may have been missed. Furthermore, CNV analysis was unavailable for WES data from five patients. Also, the access to functional genomic studies such as transcriptomics in order to verify the effect of potentially causal variants was limited. However, we have ongoing projects using long-read sequencing and RNA-sequencing to study complex genomic variants such as the structural rearrangement in patient P2605_175 and the Alu-insertion in *FGFR2* in patient P_3, respectively.

## 6 Conclusion

In summary, our findings demonstrate the power of WGS/WES as a diagnostic tool capable of generating an increased diagnostic yield in patients with unusual syndromic presentations. This is evident in view of the heterogeneity of CS and the detection of new candidate genes with pleiotropic effects. In particular, attention should be given to the phenotypic assessment of the patients and their parents, which enables a reliable interpretation of the genetic variants. Additionally, the results demonstrate the advantage of using HPO-driven variant filtration for detecting new candidate genes or variants in genes rarely associated with CS. Furthermore, the results presented a high detection rate of possibly relevant variants in patients with NCS, thereby emphasizing the need for more extensive studies, including transcriptome analysis of larger patient cohorts. Such studies will promote a broader understanding of the molecular pathogenesis of SCS as well as NCS with suggested polygenic contribution.

## Data Availability

The causal variants were submitted to ClinVar (https://www.ncbi.nlm.nih.gov/clinvar/) under the following accession numbers: 1) SUB13964571: SCV004174841 - SCV004174846; 2) SUB14012407: SCV004174877 - SCV004174882.
