# Supplementary material for: The value of genome-wide analysis in craniosynostosis
Source: Front Genet. 2024 Jan 22;14:1322462. doi: 10.3389/fgene.2023.1322462 (PMC10839781; doi:10.3389/fgene.2023.1322462)
Supplement: Supplementary file 1 [file DataSheet1.zip › Figure S3.DOCX]

Supplementary Figure 3

**Supplementary Figure 3.** Distribution of molecular outcomes according to suture pattern **(A)** in SCS **(B)** and NCS **(C)**.
