# Supplementary material for: The value of genome-wide analysis in craniosynostosis
Source: Front Genet. 2024 Jan 22;14:1322462. doi: 10.3389/fgene.2023.1322462 (PMC10839781; doi:10.3389/fgene.2023.1322462)
Supplement: Supplementary file 1 [file DataSheet1.zip › Figure S4.DOCX]

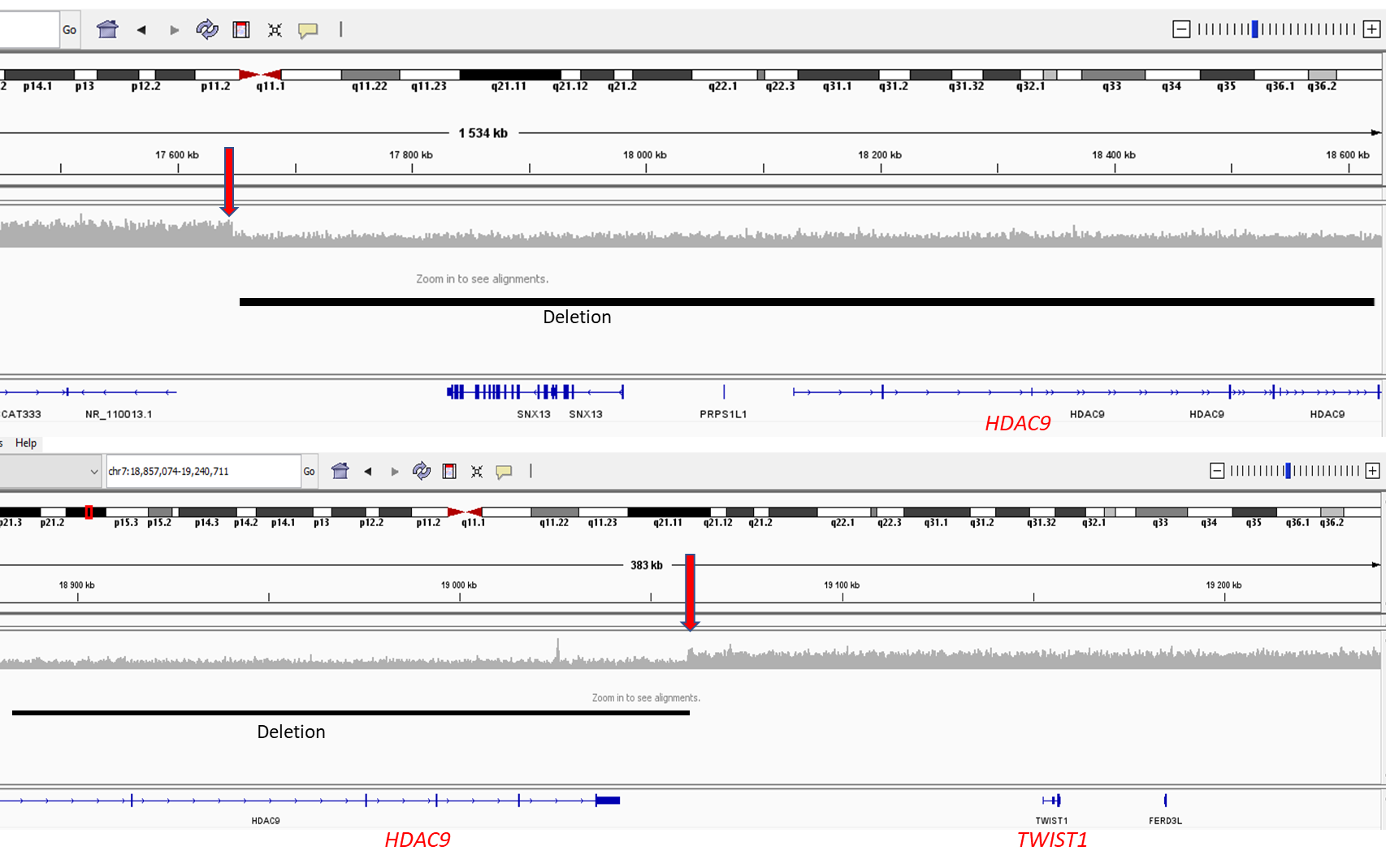
Supplementary Figure 4

Supplementary Figure 4. Screenshot from IGV showing the breakpoints of the deletion (marked with red arrows) involving HDAC9 in patient P2605_132. (Table 1).
