# Supplementary material for: The value of genome-wide analysis in craniosynostosis
Source: Front Genet. 2024 Jan 22;14:1322462. doi: 10.3389/fgene.2023.1322462 (PMC10839781; doi:10.3389/fgene.2023.1322462)
Supplement: Supplementary file 1 [file DataSheet1.zip › Methods S1.DOCX]

*Supplementary Methods 1*

**List of genes for in silico panels and additional description of the methods used for the whole-genome (WGS) and whole-exome (WES) analyses**

List of genes included in the **in silico clinical** panel (A) and **in silico research** panel (B)

1. **29 genes*:*** *ALX4, ASXL1, BMP4, CD96, CDC45, COLEC11, CYP26B1, EFNB1, ERF, FGFR1, FGFR2, FGFR3, FREM1, GLI3, IFT122, IL11RA, KMT2D (MLL2), MASP1, MEGF8, MSX2, POR, RAB23, RECQL4, SKI, SMAD6, TCF12, TWIST1, WDR35, ZIC1*
2. **133 genes:** *ABCC9, ACTB, ACTG1, ADAMTSL4, AHDC1, ALPL, ALX3, ALX4, ASXL1, ASXL3, AXIN2, BBS9, BCL11B, B3GAT3, BGN, BMP2, BMP4, BRAF, CCBE1, CD96, CDC45, CDK8, CDK13, CDKN1C, CDH11, CHD3, CHD7, CHST3, COLEC10, COLEC11, CRTAP, CYP26B1, DPH1, EFNA4, EFNB1, EHMT1, ERF, ESCO2, FBN1, FGF9, FGFR1, FGFR2, FGFR3, FLNA, FLNB, FREM1, FTO, GDF5, GLI2, GLI3, GLIS3, GPC3, GPC4, H3-3A, H3-3B, HDAC9, HNRNPK, HUWE1, IFT140, IFT122, IFT43, IGF1R, IHH, IL11RA, IL6ST, JAG1, KANSL1, KAT6A, KAT6B, KDM6A, KDM6B, KMT2D, KPTN, KRAS, LRP2, MAN2B1, MAPRE2, MASP1, MCPH1, MED12, MED13L, MEGF8, MN1, MSX1, MSX2, NFIA, NFIX, NOG, NOTCH2, NSD1, NTRK2, OSTM1, P4HB, POR, PPP1CB, PTCH1, PTH2R, PTPN11, PTPRD, RAB23, RECQL4, RSPRY1, RUNX2, SCN4A, SEC24D, SH3BP2, SHOC2, SIX1, SKI, SLC25A24, SMAD6, SMC1A, SMO, SOX6, SOX11, SON, SPECC1L, SPRY1, SPRY4, SRCAP, TCF12, TCOF1, TFAP2B, TGFBR1, TGFBR2, TUBB, TWIST1, WDR19, WDR35, ZEB2, ZIC1, ZNF462, YY1*.

**WGS via SciLifeLab (Stockholm, Sweden) and the Center of Medical Genomics (CMG) at the Sahlgrenska University Hospital**

WGS was performed at SciLifeLab (Stockholm, Sweden) on 24 patient samples. Genomic DNA was quantified using Qubit 2.0 Fluorometer (Invitrogen), fragmented to an average of 350-bp fragments using E220 focused-ultrasound sonicator (Covaris). 1 μg of fragmented DNA was thereafter converted into a sequencing ready library using TruSeq DNA PCR-free HT Sample preparation (Illumina). The library was quantified using KAPA SYBR FAST qPCR (Kapa Biosystems) and pair-end (2x150 bp) sequenced to an average of 30x coverage on the HiSeq X system (Illumina) using v2 flow cells. *Thirty* additional patient samples were analyzed by WGS at the CMG at Sahlgrenska University Hospital (SU). DNA samples were quantified using the Qubit system (Life Technologies, Carlsbad, CA, USA) and Tape Station 2200/4200 (Agilent Technologies, Santa Clara, CA, USA) for quality assessment. This quantification method was applied for the samples analyzed on a WES platform, as well. Library preps were done with TruSeq DNA PCR-free protocol (Illumina) and sequenced on NovaSeq 6000 (Illumina, 150bp x 2, pair-end reads). The bioinformatic analysis for all 54 samples was performed at SciLife Clinical Genomics Gothenburg using DNA-scope (Sention) for mapping and variant detection of Single Nucleotide Variant (SNV) and indels. For structural and copy number variation (CNV) detection the bioinformatics tools CANVAS (Illumina) and Manta (Ilumina) were used. A minimum of 97% of the genome was covered at least 10x.

**WES via the CMG at the Sahlgrenska University Hospital**

Six patient samples were analyzed by WES at the CMG at Sahlgrenska University Hospital (SU) (two patients were analyzed with both WES and WGS). Library preps were done with Agilent Sureselect Clinical Research Exome v2 and sequenced on NextSeq 500 (Illumina). Mapping and variant detection was performed at SciLife Clinical Genomics Gothenburg using CLC biomedical workbench v 4.1. (Qiagen). A minimum of 98% of the exome was covered at least 10x.
