# Supplementary material for: The value of genome-wide analysis in craniosynostosis
Source: Front Genet. 2024 Jan 22;14:1322462. doi: 10.3389/fgene.2023.1322462 (PMC10839781; doi:10.3389/fgene.2023.1322462)
Supplement: Supplementary file 1 [file DataSheet1.zip › Table S1.DOCX]

Supplementary Table 1 (phenotypic details)

**Patients with causal variants (listed in Table 1)**

| **Patient ID** | **Gender** | **Heredity for CS and other familial information (if available)** | **Nonsyndromic vs Syndromic** | **Type of CS** | **Clinically suspected diagnosis** | **Craniofacial phenotype** | **Prenatal/birth outcome** | **Neurocognitive/behavioral impairments** | **Other phenotypic data** |
| --- | --- | --- | --- | --- | --- | --- | --- | --- | --- |
| **P2603_144** | M | No | Syndromic | Bicoronal | Baraitser-Winter/Fryns-Aftimos? BPES/X-linked Ohdo-like? Saethre-Chotzen/Muenke/Marfan/Loeys-Dietz syndrome | Broad face, ptosis, hypertelorism, telecanthus, epicanthus inversus, downslanting palpebral fissures, short and broad nose with anteverted nares, long philtrum |  | Gross motor delay, impaired balance and coordination, learning disability, attention deficit disorder. | Pulmonary stenosis, cryptorchidism, pectus excavatum, bilateral inguinal hernia, dilated aortic root and ascendent aorta, short and broad neck, low posterior hairline (trident), iris coloboma, high and narrow palate |
| **P2605_105** | F |  | Syndromic | Right coronal | Saethre-Chotzen syndrome -like | Prominent forehead, bilateral epicanthus, S-formed blepharoptosis, short and downslanting palpebral fissures, low-set dysplastic posteriorly rotated ears with prominent crus, beaked nose, maxillary hypoplasia |  | High intracranial pressure | Cleft soft palate, absent uvula, irregular teeth, high hard palate with bilateral grooves, narrow auditory canal, nl growth |
| **P2605_115** | M |  | Syndromic | Sagittal + lambdoid bilateral (Mercedes synostosis) | Catel-Manzke-like appearance, but finger abnormalities? | High forehead, flat occipital region, lateral eyebrow flare/upsweep, short palpebral fissures, low-set posteriorly rotated ears, malar flattening, microstomia and microretrognathia | Caesarean section because of preeclampsia at 37gw, high birth weight | Microcephaly (-4SD), neuronal migration defect, speech delay | Hypospadias, abnormal aortic arch, growth delay (?) |
| **P2605_102** | M |  | Syndromic | Bicoronal+metopic+ sagittal (?) | Atypical Shprintzen-Goldberg syndrome | Bulging fronto-temporal regions, flat malar regions, pointed head, dysplastic small and low-set ears, shallow orbits, short palpebral fissures, large nasal tip, microretrognathia | Born 10 days before term, birth weight 2820g, length 47 cm, OFC=33,8 cm, asphyxia (Apgar 5-6-8), hypoglycemia | High intracranial pressure, developmental delay including speech (nonverbal at 14 years old) | Kyphoscoliosis (corset), pectus carinatum, long and thin fingers (possibly arachnodactyly), hypermobile finger joints and wrists, long toes, atrial septal defect (op.) and regurgitating mitral valve prolaps, hearing loss, cryptorchidism |
| **P2605_132** | F |  | Syndromic | Right coronal | Saethre-Chotzen-like at a later age. Branchio-otic syndrome (BOS)? | Short palpebral fissures (blepharophimosis), epicanthus with telecanthus, malar flattening, low-set small ears with overfolded helix and underdeveloped connection between tragus and antitragus, prominent crus |  | Possibly speech delay at 3 years old, otherwise normal dev. | Narrow auditory channels, bilateral sensorineural hearing loss, left branchial cyst. Normal growth at 3 years old. |
| **P2605_175** | F |  | Syndromic | Right coronal | Saethre-Chotzen syndrome | Turricephaly, asymmetric face, flattened frontotemporal region with right sided plagiocephaly, sloping forehead, left ptosis, prominent nasal pyramid, hypoplastic/notched alae naesi with relatively prominent columella, flat malar regions, small ears with short earlobe, microretrognathia, broad philtrum | Caesarean section 42gw |  | Divergent strabismus (trochlear nerve paresis), bilateral mild sensorineural hearing loss, sacral hair, high palate, ASD, VSD, long fingers, flexed left big toe |
| **P_1** | F | Mother's cousin operated for sagittal synostosis | Syndromic | Bicoronal |  | Turribrachycephaly, broad forehead, hypertelorism, short palpebral fissures, short nose with broad nasal bridge, small nasal tip with hypoplastic notched alae naesi, short philtrum, tented upper lip vermillion with downturned corners of the mouth, high palate, low-set dysplastic ears with prominent ear crus and uplifted ear lobe | Uneventful pregnancy, born at 41+2 gw, BW=4640g | Normal early development, tendency to keep the index fingers flected | Accessory mamilla on the right, simian crease on the left hand. At 6 months of age, before operation for CS: OFC=46,1 cm (+2,6SD), W=9680g (+2,3SD), L=68 cm (+0,6SD). |
| **P_2** | F |  | Syndromic | Sagittal synostosis |  | Macrocephaly (OFC=53,9 cm, +2,5SD after operated CS at 3,5y), typical scaphocephaly with frontal bossing and prominent occiput, broad forehead, hypertelorism with short palpebral fissures, epicanthus, mild unilateral right ptosis, short nose with broad and flat nasal bridge, small alae naesi, tented upper lip vermillion and open mouth, low-set ears | Uneventful pregnancy and delivery at term (39+1 g.w), mother treated with Citalopram and Levaxin during pregnancy. | Psychomotor delay, walked at 2 years old, speech delay, focal seizures with debut at 2,5 years old. Brain MRI at 3,5 y - slightly reduced white matter laterally to the occipital horns of the lateral ventricles |  |
| **P_3** | F |  | Syndromic | Bicoronal + lambdoid(?) | Apert (typical) | Cloverleaf skull with bone defects, hypertelorism, proptosis, midface hypoplasia, short nose with flat nasal root, high palate, small open mouth with short philtrum, short neck | Placenta previa (bleedings) | Delayed psychomotor development, 2 seizure attacks, general hypotonia | Total bilateral syndactyly of fingers ("mitten hands") and toes with synonichia, possibly glenohumeral dysplasia (dimples on shoulders), strabismus, sleep apnea (need of CPAP), recurrent infections, broad thorax with wide spaced mammillae, tendency to thoracolumbar kyphosis, limited shoulder and elbow extension |
| **P_4** | M |  | Syndromic | Sagittal synsotosis |  | Scaphocephaly with frontal bossing, prominent occiput, parietal narrowing, hypertelorism, upslanting palpebral fissures, low-set ears with overfolded helix, short nose, broad and depressed nasal root, downturned corners of the mouth, small chin, microretrognathia | Born at 40+6 g.w following uneventful pregnancy, mother treated with Levaxin | Delayed speech and fine motor development, feeding difficulties, autistic features. No clear signs of high ICP. Brain MRI: marked lateral ventricles and frontoparietal cortical convolutions, slightly increased liquor surrounding the optic nerves. |  |
| **P_5** | F | No, but the patient's facial treats remind of her mother (hypertelorism) | Syndromic | Sagittal | Possibly Crouzon (atypical) | Scaphocephaly with frontal bossing and prominent occiput, broad forehead, biparietal narrowing, proptosis, mild hypertelorism or rather epicanthus with telecanthus, flat nasal root, short nose with anteverted nares, low-set ears with overfolded helices, downturned corners of the mouth, round face. | Gestational diabetes, vaginal birth (35+5 g.w) | Hypotonia (?) |  |

**Patients with SCS and VUSs (listed in Supplementary Table 3)**

| **Patient ID** | **Gender** | **Heredity for CS and other familial information (if available)** | **Nonsyndromic vs Syndromic** | **Type of CS** | **Clinically suspected diagnosis** | **Craniofacial phenotype** | **Prenatal/birth outcome** | **Neurocognitive/behavioral impairments** | **Other phenotypic data** |
| --- | --- | --- | --- | --- | --- | --- | --- | --- | --- |
| **P2605_155** | M |  | Syndromic (?) | Right coronal+frontoshenoidal + metopic synostosis | Crouzon-like | High forehead with high frontal hairline, prominent metopic suture, shallow orbits, flat malar regions, short nose, long philtrum, small mouth, thin lips, low-set and posteriorly rotated ears, prominent left ear helix, right preauricular pit | Nl, vaginal birth at term, vacuum extraction | A priori normal psychomotor development | High and narrow palate |
| **P2605_104** | M |  | Syndromic | Pansynostosis | Frontonasal dysplasia, Sweeney-Cox-like, Pfeiffer syndrome | Unilateral(?) cloverleaf skull, peaked head, paramedial left midline raphé with frontonasal dysplasia, bifid nasal tip, dysplastic/hypoplastic alae naesi, severe hypertelorism, short palpebral fissures, flat malar regions, low-set ears |  | Hydrocephalus | High, angulated and narrow palate, hypertrophic gums, nasal speech, left choanal atresia, strabismus, astigmatism with hypermetropia, scoliosis, monoarthritis left knee, pulmonary hypertension |
| **P2603_114** | M |  | Syndromic | Sagittal + bicoronal (partial) + left lambdoid | ? | Bulging forehead before surgery, then turricephaly with high forehead, blepharophimosis, epicanthus inversus, telecanthus, proptosis, short nose with flat nasal root, low-set ears (asymmetric because of the skull deformation), flat malar regions, microstomia |  |  | Abnormal iris morphology (grey spots) |
| **P2605_168** | M |  | Syndromic | Bicoronal | Saethre-Chotzen syndrome | Brachycephaly, low frontal hairline with widow's peak, prominent and broad nasal bridge, short and downslanting palpebral fissures, discrete epicanthus, flat malar regions, low-set and dysplastic ears with angulated helix and hypoplastic attached earlobe, absent tragus, mild microretrognathia |  |  | Skin syndactyly on fingers, funnel chest, Gilberts syndrome (unconjugated hyperbilirubinemia) |
| **P2605_136** | M | No | Syndromic | Metopic+sagittal | Carpenter syndrome-like type 2? Gorlin-Chaudhry-Moss sd.? | Trigonocephaly, scaphocephaly, turricephaly after operation, hypertelorism with telecanthus, arched eyebrows, shallow orbits, broad and flat nasal bridge and tip, thick alae naesi, small, pointed chin, low-set, posteriorly rotated and dysplastic ears with almost unfolded helix | Nl, vaginal birth at term | Partial corpus callosum agenesia, mega cisterna magna, mild developmental delay | Severe bilateral hearing loss with inner ear malformation - Mondini dysplasia, myopia, hypogonadism with cryptorchidy, hypoplastic scrotum, apnea, pectus carinatum, thoraco-lumbar kyphosis and scoliosis, cutaneous syndactyly 2-5th fingers bilateral and 2nd-3rd toes, camptodactyly of fingers 3-5 right and 3-4 left, short middle phalanges, camptodactly of toes 4-5, large big-toes, flat feet, genu valgum, high palate, persistent deciduous teeth, solitary median maxillary central incisor, iron deficiency anemia, operated finger neurofibroma |
| **P2605_134** | F | No | Syndromic (?) | Right coronal+sagittal+lambdoid bilateral |  | Frontal bossing (in infancy, before surgery), high forehead scaphocephaly combined with brachyturricephaly, asymmetric face, discrete epicanthal folds (in infancy), short palpebral fissures, malar flattening | Gestational diabetes (3rd trimester), congenital synostosis | Speech delay | Strabismus |
| **P2605_175** | F |  | Syndromic | Right coronal | Saethre-Chotzen syndrome | Turricephaly, asymmetric face, flattened frontotemporal region with right sided plagiocephaly, sloping forehead, left ptosis, prominent nasal pyramid, hypoplastic/notched alae naesi with relatively prominent columella, flat malar regions, small ears with short earlobe, microretrognathia, broad philtrum | Caesarean section 42gw |  | Divergent strabismus (trochlear nerve paresis), bilateral mild sensorineural hearing loss, sacral hair, high palate, ASD, VSD, long fingers, flexed left big toe |
| **P2605_157** | F |  | Syndromic | Left coronal + metopic (?) | | High forehead, tendency to turricephaly preoperative, prominent scalp veins, shallow orbits, mild right proptosis, flat malar regions, small mouth, hypotelorism, asymmetric facial midline, right epicanthus, low-set and prominent ears (anteriorly rotated) and cup-shaped right helix | Nl, vaginal | Gross motor delay (walk independently at 19 months of age) | Unilateral convergent strabismus, tapering fingers, mild skin syndactyly of fingers II-II-IV, short proximal phalanx II-V, bilateral syndactyly of toes II-III, overlapping IInd and IVth toes right foot, clinodactyly of IIIrd toe, diarrhea, gastroesophageal reflux, food allergy, growth delay, short stature, sparse hair |
| **P2605_166** | M | Father has hearing loss, syndactyly of toes II-III, and seborrheic eczema; mother has dysplastic helix and syndactyly of toes II-III; maternal grand-mother operated for ptosis | Syndromic (?) | Left coronal+ left lambdoid | Saethre-Chotzen syndrome ? | Asymmetric face and skull, left plagiocephaly, right ptosis, blepharoptosis, dysplastic helix (overfolded), flat malar regions, prominent nasal pyramid |  | Autistic features, ADHD | Syndactyly of toes II-III, congenital torticollis, ventricular septal defect (VSD) |
| **P_10** | M | No | Syndromic (?) | Right unicoronal |  | Right plagiocephaly, mild facial asymmetry, low-set ears with prominent crus and overfolded helix, short nose, everted lower-lip. |  | Delayed speech at 3 years old, hyperactivity | Mild unilateral convergent strabismus |
| **P_13** | M | No | Syndromic | Bicoronal + sagittal |  | Bitemporal narrowing, proptosis, low-set ears, malar flattening, short nose with flat nasal tip and short columella (possibly an ethnic feature), microretrognathia | Premature (25+1 g.w) caesarean section, Apgar 7-9-10, BW=760g, assisted ventilation | Possibly mild psychomotor delay, MRI: Chiari type 1, narrow foramen magnum | Bronchopulmonary dysplasia (BPD of prematurity), inguinal hernia (op.), growth delay (At 15m - L=72 cm (-3SD), W=8260g (-3SD). At 2y - W=11,5Kg (-1,6SD) |
| **P2605_186** | M | Father has mild syndactyly of toes II-III; possibly right ptosis in a paternal uncle; mother had cervical branchial fistula | Syndromic (?) | Right coronal + lambdoid? | Saethre-Chotzen syndrome-like | Turricephaly with right plagiocephaly, mild frontal bossing, s-formed left ptosis, downslanting palpebral fissures, broad nasal bridge, low-set ears | Caesarian section (placenta previa) |  | Strabismus (op.), sleep apnea, cervical branchial fistula (as mother) |
| **P_15** | F | No | Syndromic | Sagittal + lambdoid bilat (Mercedes synostosis) |  | Scaphocephaly with frontal bossing, malar flattening, small chin, thin upper lip vermillion and everted lower lip, low-set and posteriorly rotated ears |  |  |  |
| **P_21** | F |  | Syndromic | Bicoronal |  | Brachycephaly, round face with hirsutism (possibly secondary to Proglycem treatment), low anterior hairline, horizontal long eyebrows with synophrys, long eyelashes, short nose with flat nasal bridge, mild epicanthus, triangular mouth with downturned corners, short philtrum, thin upper lip vermillion, low-set ears with overfolded helix, short neck | Born at 39+3 g.w, BW=3685g (+0,4SD), BL=48 cm (-1SD), OFC=33,5 cm (-1SD). Neonatal hypoglycemia. | Mild developmental delay | Hyperinsulinism with recurrent hypoglycemia treated with Proglycem. Facial, hand, legs hirsutism (possibly secondary effect to Proglycem). |
| **P_18** | F | Mother had recurrent miscarriages | Syndromic(?) | Right unicoronal |  | Prominent forehead and glabella, facial asymmetry with right plagiocephaly, low-set ears, small mouth with downturned corners, tented upper lip vermillion, high palate, short and small nose. | Uneventful pregnancy, born at 38+2gw. | Discrete head-lag at 4m; satisfactory psychomotor dev. at 3 years old | Intermittent mild strabismus |
| **P_16** | F |  | Syndromic | Left unicoronal |  | Left plagiocephaly, brachycephaly, broad face, pointed chin, mild facial midline scoliosis with orbital asymmetry, hypertelorism, short and downslanting palpebral fissures with telecanthus, short nose, thin upper lip vermillion, downturned corners of mouth with everted lower lip vermillion, possibly mild mandibular prognathism |  | ? | Torticollis |

**Patients with NCS and VUSs (listed in Supplementary Table 4)**

| **Patient ID** | **Gender** | **Heredity for CS and other familial information (if available)** | **Nonsyndromic vs Syndromic** | **Type of CS** | **Craniofacial phenotype** | **Prenatal/birth outcome** | **Neurocognitive/behavioral impairments** | **Other phenotypic data** |
| --- | --- | --- | --- | --- | --- | --- | --- | --- |
| **P_6 (Quatro)** | M | Mother and brother with CS | Nonsyndromic (?) | Sagittal + partial bicoronal synostosis | Brachycephaly with bitemporal narrowing and biparietal bossing, even anterior sagittal bossing, flat occiput, relatively short palpebral fissures, short/small nose, short philtrum, small mouth, small chin (microretrognathia), low-set and posteriorly rotated ears | Born at 41 g.w by vacuum extraction |  |  |
| **P_7 (Quatro)** | M | Mother and brother with CS | Nonsyndromic (?) | Sagittal synostosis | Dolichocephaly with prominent occiput, mild bitemporal narrowing, mild shortening of palpebral fissures with mild epicanthus, short nose, flat nasal bridge, small mouth and chin (microretrognathia), low-set and posteriorly rotated ears | Uneventful pregnancy and delivery at term. |  |  |
| **P_8 (Quatro)** | F (P_6 and P_7's mother) | Two sons with CS | Nonsyndromic (?) | Sagittal synostosis | Scaphocephaly with bitemporal narrowing | Uneventful pregnancy, born at 40 g.w, BW=3200g |  |  |
| **P2605_113** | F |  | Nonsyndromic | Bicoronal | Brachyturricephaly, bone defect at the level of the coronary suture, low anterior hairline, Crouzon-like at birth, but not at a later age. No obvious dysmorphic features in adulthood unless very low anterior hairline. | Crouzon-like at birth |  |  |
| **P2603_129** | F |  | Nonsyndromic (?) | Right coronal | High forehead, low-set ears, posteriorly rotated, short nose, long and smooth philtrum, thin lips, small mouth | Caesarian section at 41+5gw | Hyperactive (according to parents) |  |
| **P2605_195** | M |  | Nonsyndromic (?) | Right coronal | Facial asymmetry, unilateral right epicanthus, downslanting palpebral fissures. |  |  | Heart murmur, mild vision loss on the right eye (myopia/ectopia lentis?) |
| **P2603_156** | M |  | Nonsyndromic | Sagittal+bicoronal partial | Scaphocephaly at initial assessment, bitemporal narrowing, overfolded helix, low-set ears, short, small nose, thin lips | Breech position, vaginal birth at term |  |  |
| **P2605_170** | M |  | Nonsyndromic | Right coronal + sagittal | Short palpebral fissures with mild telecanthus, anteriorly rotated ears, flat malar regions |  |  |  |
| **P2605_150** | M | Distant consanguinity, father has unilateral 2-3 toe syndactyly, syndactly in maternal cousin | Nonsyndromic (?) | Right coronal | Brachycephaly, flattened forehead, mild nasal scoliosis, mild left blepharoptosis, low-set posteriorly rotated ears |  | Strabismus, delayed visual development | 2-3 bilateral toe syndactyly, tight neck muscles |
| **P2605_181** | F |  | Nonsyndromic (?) | Sagittal + lambdoid bilateral (partial) | Prominent occiput, tendency to scaphocephaly, discrete epicanthus with telecanthus, relatively short palpebral fissures, short and small nose with broad nasal root and prominent nasal bridge, anteverted nares, short columella, long philtrum, thin upper lip, small mouth |  | High intracranial pressure (cerebellar herniation) | Neonatal hypoglycemia |
| **P_12** | F | No | Nonsyndromic | Right unicoronal | Right plagiocephaly, mild facial midline scoliosis with orbital asymmetry, short palpebral fissures, discrete epicanthal folds, malar flattening, short nose with flat nasal root, small mouth | Born at 38 g.w, BW=2900g, scheduled caesarean section. Mother had diabetes during pregnancy, treated with insulin. |  |  |
| **P_11** | F | No | Nonsyndromic (?) | Left unicoronal | Mild epicanthal fold/telecanthus, slightly asymmetric orbitae with mild left ptosis, short nose with flat nasal root, small mouth | Born at 40+6 g.w (vacuum extraction) |  |  |
| **P2605_174** | F |  | Nonsyndromic | Right coronal | Low anterior hairline, asymmetric face, flat malar regions, low-set and posteriorly rotated right ear | 40gw | Normal development | Strabismus |
| **P2603_135** | F |  | Nonsyndromic (?) | Left coronal | Flat malar regions, low set anteriorly rotated (in infancy) and relatively small ears |  | Normal development in infancy | Strabismus, torticollis |
| **P2605_163** | M | No, father and paternal grandfather have a mild 2-3 toe syndactyly | Nonsyndromic (?) | Left coronal | Brachycephaly, low-set ears, mild epicanthus, small, short nose, flat malar regions, small mouth |  |  | Right sided torticollis , mild 2-3 toe syndactyly, strabismus |
| **P2605_183** | F |  | Nonsyndromic | Left coronal | Plagiocephaly, open mouth, no obvious dysmorphic features, discrete facial asymmetry | 38gw | Normal development | Torticollis |
| **P2603_163** | F |  | Nonsyndromic | Left coronal+ispilateral frontosphenoid | Asymmetric skull and position of the eyes (facial scoliosis), especially as a baby, less evident at a later age: discrete epicanthus, short, small nose with flattened wide nasal root, small mouth | Nl birth, cephalohematoma, asymmetric skull | Normal development at 5 years of age |  |
| **P2605_189** | M |  | Nonsyndromic (?) | Right coronal | No photos available |  | Asperger syndrome, psychiatric and possibly cognitive problems | Strabismus |
| **P2603_119** | M |  | Nonsyndromic | Right coronal+partial sagittal | Scaphocephaly with right plagiocephaly in infancy (preop.), short palpebral fissures, discrete epicanthus with telecanthus (in infancy) | Premature rupture of membranes-PRM (4 days), prolonged delivery=> caesarian section (CS), Nl BW,BL and OFC, but asymmetric skull | Normal development |  |
| **P_22** | M |  | Nonsyndromic | Right unicoronal | Right plagiocephaly, orbital asymmetry, facial midline scoliosis, low-set ears |  |  |  |
| **P_19** | F | No | Nonsyndromic | Right unicoronal | Mild facial asymmetry with right plagiocephaly, limit low-set ears. | Uneventful pregnancy, born at 42gw by caesarean section. |  | Thalassemia |
| **P_20** | F |  | Nonsyndromic (?) | Right unicoronal | Facial asymmetry with right plagiocephaly, limit low-set ears, short nose, flat nasal root, discrete epicanthus with slightly downslanting palpebral fissures, though no general dysmorphic appearance, postop. tendency to brachycephaly. | Born at 39+6gw. |  |  |
| **P2603_161** | F |  | Nonsyndromic | Right coronal | Discrete facial asymmetry and nasal deviation | Preterm (2 g.w) |  |  |
| **P2605_187** | F | Elder brother same phenotype | Nonsyndromic (?) | Sagittal | Scaphocephaly, high and prominent forehead and occiput, short palpebral fissures, small and low-set ears with overfolded helix, short nose, small mouth |  | ? |  |
| **P2603_153** | F |  | Nonsyndromic | Left coronal | Mild orbital asymmetry, discrete malar flattening, small chin | Uneventful, asymmetric skull |  |  |
| **P2603_152** | M |  | Nonsyndromic | Left coronal + sagittal | High forehead with bitemporal narrowing, short/small nose, low-set ears with prominent ear crus | Prematurity 26+6gw (twin pregnancy), BW=835g | CT-digital impression | Right inguinal hernia (op.) |

**Patients with negative results at WES/WGS (whereof one - P2603_151 - with a previously reported VUS from NGS targeted analysis, but no additional findings at WGS)**

| **Patient ID** | **Gender** | **Heredity for CS and other familial information (if available)** | **Nonsyndromic vs Syndromic** | **Type of CS** | **Clinically suspected diagnosis** | **Craniofacial phenotype** | **Prenatal/birth outcome** | **Neurocognitive**  **behavioral impairments** | **Other phenotypic data** | **Previous clinical and/or research analyses** | **Genes with previously published variants** |
| --- | --- | --- | --- | --- | --- | --- | --- | --- | --- | --- | --- |
| **P2603_151** | F |  | Syndromic | Right coronal | Klippel-Feil with features of MURCS (Mullerian duct aplasia, unilateral renal aplasia, and cervicothoracic somite dysplasia) | Prominent nasal root and ridge, short nose with prominent columella and small alae naesi, synophrys, small mouth with downslanting corners, small and low-set ears |  |  | Short neck, possibly fused upper thoracic vertebrae, possibly small accessory cervical rib left, uterus aplasia, horseshoe kidney (mild variant), several pigmented nevi on the face and neck | Target research panel (63 genes) | PTCH1 (Topa et al., 2020) |
| **P_14** | M |  | Syndromic (?) | Bicoronal | Saethre-Chotzen (atypical) | Brachycephaly, low anterior hairline, broad forehead, malar flattening, short palpebral fissures, short nose, discrete anteversion of ears and overfolded helix |  |  |  | In silico panel WGS (29 genes) + MLPA |  |
| **P_9** | M |  | Syndromic | Right unicoronal |  | Facial asymmetry, small face, low-set ears with overfolded helix, mild proptosis, epicanthus, malar flattening, with short columella, small mouth | Uneventful pregnancy, born at 39+2gw by caesarean section for breech. | Psychomotor delay, walked at 16m, clumsiness, speech delay, autistic features | Large hands, tapering fingers (?) | Microarray, in silico panel WES (29 genes) + MLPA, |  |
| **P2605_110** | F |  | Nonsyndromic (?) | Bicoronal |  | High forehead, flat malar regions, discrete epicanthus, downslanting palpebral fissures, mild left ptosis, small ears | Caesarean section | Normal cognitive dev. |  | Target research panel (63 genes) |  |
| **P2605_145** | F |  | Nonsyndromic | Right coronal |  | Discrete epicanthus, low-set and slightly dysplastic left ear (prominent crus) |  |  |  | Target research panel (63 genes) |  |
| **P2605_156** | M | Elder sister - same diagnosis | Nonsyndromic | Sagittal |  | Scaphocephaly, prominent forehead, slightly bulging on the right side, prominent occiput, very mild left parietal bulging, right epicanthus, microretrognathia |  | CT-3D: asymmetric lateral ventricles (R>L), periventricular leukomalacia right |  | Target research panel (63 genes) |  |
| **P_17** | F | No | Nonsyndromic | Right unicoronal |  | Mild facial asymmetry with right plagiocephaly, mildly low set anteverted ears (not dysplastic) | Born by caesarean section at 38gw |  |  | In silico panel WES (29 genes) + MLPA |  |
