# Supplementary material for: The value of genome-wide analysis in craniosynostosis
Source: Front Genet. 2024 Jan 22;14:1322462. doi: 10.3389/fgene.2023.1322462 (PMC10839781; doi:10.3389/fgene.2023.1322462)
Supplement: Supplementary file 1 [file DataSheet1.zip › Table S2.DOCX]

Supplementary Table 2

**Secondary findings with possible modulator effect in CS-relevant genes detected in patients with causal variants (Table 1).**

| **Patient no (gender)** | **Sutural pattern** | **Phenotype** (clinically suspected diagnosis) | **Gene** (transcript) | **Variant annotation - cDNA, protein level** | **Variant classification according to ACMG criteria** (novelty, zygosity, inheritance, molecular aspects^1^) | **Detection by screening methods** | | **Relevant associated disorder** (OMIM, PubMed – PMID, Inheritance pattern) |
| --- | --- | --- | --- | --- | --- | --- | --- | --- |
|  |  |  |  |  |  | **In-silico panel on WGS/WES** (133 genes) | **HPO-term analysis with Moon/Alissa software** |  |
| **P2603_144 (M)** | Bicoronal | SCS (Noonan, Loeys-Dietz, Baraitser-Winter, BPES, Ohdo-like) | ***IFT81***  NM_014055.3 | c.1217G>A, p.(Ser406Asn) | VUS (het, carrier, maternal, no other variant detected)  0.079868% (111) in gnomAD, missense, moderately conserved, small physicochemical difference, 4/4 tolerated, reported as VUS in ClinVar (VCV000848922.3) | - | + (only Moon) | Short-rib thoracic dysplasia 19 with or without polydactyly (#617895, AR) |
|  |  |  | ***POR***  NM_001382657.1 | c.683C>T, p.(Pro228Leu) | VUS (het, carrier, maternal, no other variant detected)  0.49548% (687 - het) and 0.00072122% (1 - hom) in gnomAD, missense, highly conserved, moderate physicochemical difference, reported in ClinVar (VCV000436385.21) with conflicting interpretations | + | + (only Alissa) | Antley-Bixler syndrome with genital anomalies and disordered steroidogenesis (#201750, AR) |
| **P2605_105 (F)** | Unicoronal right | SCS (Saethre-Chotzen) | ***TAOK1***  NM_020791.2 | c.2800G>T, p.(Gly934Cys) | VUS *(novel)*  (het, paternal)  Absent in gnomAD, missense in protein domain, highly conserved, large physicochemical difference, 3/4 damaging, inherited from unaffected parent | - | + | Developmental delay with or without intellectual impairment or behavioral abnormalities (#619575, AD) |
|  |  |  | ***CHD5***  NM_015557.2 | c.3545G>A, p.(Gly1182Glu) | VUS *(novel)*  (het)  Absent in gnomAD, missense in protein domain, highly conserved, moderate physicochemical difference, 4/4 damaging, alternative molecular basis for the disease, assumed inherited from unaffected parent | - | + (only Moon) | Parenti-Mignot neurodevelopmental syndrome (#619873, AD) |
| **P2605_132 (F)** | Unicoronal right | SCS (Saethre-Chotzen-like, Branchiootic – like) | ***FREM1***  NM_144966.5 | c.3592G>A, p.(Asp1198Asn) | VUS (het)  0.0063694% (1) in gnomAD, missense, weakly conserved, small physicochemical difference, 4/4 tolerated, alternative molecular basis for disease, assumed inherited from unaffected parent | + | - | Trigonocephaly 2 (#614485, AD) |
|  |  |  | ***CDC45***  NM_001178010.2 | c.548C>G, p.(Ser183Cys) | VUS (het, carrier, no other variant detected)  0.51267% (475 – het) and 0.0021586% (2 – hom) in gnomAD, weakly conserved, moderate physicochemical difference, 1/4 damaging, reported as likely benign in ClinVar (VCV000716032.7) and pathogenic in combination with 22q11 deletion in HGMD (CM205377) | - | + (only Alissa) | Meier-Gorlin syndrome 7 (#617063, AR) |
| **P2605_175 (F)** | Unicoronal right | SCS (Saethre-Chotzen-like) | ***OGDHL***  NM_018245.3 | c.2596del, p.(Ser866Alafs*5) | VUS (het, carrier, no other variant detected)  0.0080611% (10) in gnomAD, truncating frameshift | - | + (only Moon) | Yoon-Bellen neurodevelopmental syndrome (#619701, AR) |
| **P_1 (F)** | Bicoronal | SCS | ***TLK2***  NM_001375269.1 | c.36_42dup, p.(Arg15Glyfs*41) | VUS (het)  0.051487% (8) in gnomAD, truncating frameshift, alternative molecular basis for disease, assumed inherited from unaffected parent (frequency in gnomAD) | - | + (only Alissa) | Intellectual developmental disorder, autosomal dominant 57 (#618050, AD) |
| **P_2 (F)** | Sagittal | SCS | ***MSX2***  NM_001363626.2 | c.375G>T, p.(Pro125=) | VUS (het, paternal)  Absent in gnomAD, synonymous, not conserved nucleotide, no splicing impact predicted, inherited from unaffected parent, reported as VUS in ClinVar (VCV000211523.5) | + | + (only Alissa) | Craniosynostosis 2 (#604757, AD) |
|  |  |  | ***OGDHL***  NM_018245.3 | c.2766C>G,  p.(Phe922Leu) | VUS (het, carrier, maternal, no other variant detected)  0.00086845% (1) in gnomAD, missense in protein domain, highly conserved, small physicochemical difference, 4/4 damaging | - | + (only Moon) | Yoon-Bellen neurodevelopmental syndrome (#619701, AR) |
| **P_3 (F)** | Bicoronal + lambdoid (?)  Sagittal  Sagittal | SCS (Apert, typical)  SCS  SCS (Crouzon, atypical) | ***AXIN2***  NM_004655.3 | c.1741C>T, p.(Arg581Cys) | VUS (het)  0.00082729% (1) in gnomAD, missense, highly conserved, large physicochemical difference, 3/4 damaging, reported as VUS in ClinVar (VCV000939295.3), alternative molecular basis for the disease, assumed inherited from unaffected parent. | + | + | Oligodontia-colorectal cancer syndrome (#608615, AD) |
|  |  |  |  |  |  |  |  | Sagittal synostosis, *AXIN2*-related (PMID: 34134783, AD) |
|  |  |  | ***EFNA4***  NM_005227.3 | c.583C>T, p.(Leu195Phe) | VUS (het)  0.00079587% (1) in gnomAD, missense, moderately conserved, small physicochemical difference, 2/4 damaging, alternative molecular basis for the disease, assumed inherited from unaffected parent. | + | - | *EFNA4* in syndromic forms of CS (PMID: 29168297, AD) |

^1^Genotype frequency in control population (gnomAD), effect at protein level, location in protein domain, nucleotide/amino acid evolutionary conservation, physiochemical difference between amino acids, no/4 – no of in silico prediction programs assessing the variant as damaging/tolerated per total no of programs – 4: SIFT, MutationTaster, PolyPhen-2: HumDiv and HumVar.

“-“ no detection; “+” variant detected by method; ClinVar – Clinical Genome Resource (database of variants associated with human disease); F – female; gnomAD - The Genome Aggregation Database; het – heterozygous; hom – homozygous: M – male; MLPA - multiplex ligation-dependent probe amplification; VUS – variant of uncertain significance.
