# Supplementary material for: The value of genome-wide analysis in craniosynostosis
Source: Front Genet. 2024 Jan 22;14:1322462. doi: 10.3389/fgene.2023.1322462 (PMC10839781; doi:10.3389/fgene.2023.1322462)
Supplement: Supplementary file 1 [file DataSheet1.zip › Table S3.DOCX]

Supplementary Table 3

Possibly relevant variants (including modulator effect) observed in patients with SCS and no detected causal variants explaining the CS.

| **Patient no (gender)** | **Sutural pattern** | **Phenotype** (clinically suspected diagnosis) | **Analyses prior to inclusion in the study** (on both clinical and research basis) | **Gene**  (transcript) | **Variant annotation - cDNA, protein level/genomic position for CNVs** | **Variant classification according to ACMG criteria** (novelty, zygosity, inheritance, molecular aspects^1^) | **Detection by screening method** | | | **Associated relevant disorder** (OMIM, PubMed – PMID, Inheritance pattern) | **Additional variants -**  **previously published^2^ confirmed by WES/WGS** and new discarded variants (Table S6) |
| --- | --- | --- | --- | --- | --- | --- | --- | --- | --- | --- | --- |
|  |  |  |  |  |  |  | **In-silico panel on WGS/WES** (133 genes) | **HPO-term analysis with Moon/Alissa software** | **CNV analysis** (Alissa + IGV) |  |  |
| **P2605_136 (M)** | Metopic + sagittal | SCS (Carpenter-like) | Karyotype + FISH 22q11, targeted NGS panel (63 genes) | ***ATRX***  NM_000489.5 | c.690T>G, p.(Ile230Met) | VUS *(novel)*  (hemizygous, maternal)  Absent in gnomAD, missense in protein domain, highly conserved, small physicochemical difference, 3/4 damaging | - | + (only Moon) | - | Carpenter-like, *ATRX*-related (PMID: 34348791, X-linked) | ***AXIN2, RUNX2* (**paternal),  *SETD1B* (Table S6) |
|  |  |  |  | ***TRAF7***  NM_032271.2 | c.331G>A, p.(Glu111Lys) | VUS (het, maternal)  0.0063828% (1) in gnomAD, missense, highly conserved, small physicochemical difference, 2/4 damaging, inherited from unaffected parent | - | + (only Moon) | - | Cardiac, facial, and digital anomalies with developmental delay (#618164, AD) |  |
| **P2605_157 (F)** | Left coronal + metopic (?) | SCS | HaloPlex NGS panel (12 genes) + MLPA, NGS panel (63 genes) | ***LTBP1***  NM_206943.4 | c.1737_1739del, p.(Glu579del) | VUS *(novel)* (het, carrier, no other variant detected)  Absent in gnomAD, in-frame deletion in protein domain. | - | + (only Moon) | - | Cutis laxa, autosomal recessive, type IIE (#619451, AR) | ***ERF, CYP26B1***(het, carrier, no other variant detected) |
| **P2605_166 (M)** | Left coronal + left lambdoid | SCS (Saethre-Chotzen-like)^3^ | *TWIST1* (Targeted Sanger+MLPA), target research panel (63 genes) | ***C2CD3***  NM_001286577.1 | c.3745C>T, p.(Arg1249*) | VUS *(novel)* (het, carrier, no other variant detected)  Absent in gnomAD, truncating. | - | + (only Moon) | - | Orofaciodigital syndrome XIV (#615948, AR) | ***MSX1*** |
| **P2605_155 (M)** | Unicoronal right + metopic+ frontosphenoidal | SCS^3^  (Crouzon-like) | Targeted NGS panel (63 genes) | ***ZNF462***  NM_021224.6 | c.1832C>A, p.(Thr611Asn) | VUS (het)  0.0071573% (9) in gnomAD, missense in protein domain, moderately conserved, small physicochemical difference, 3/4 damaging, assumed inherited from unaffected parent (frequency in gnomAD) | + | + | - | Weiss-Kruszka syndrome (#618619, AD) | ***CYP26B1*** (het, carrier, no other variant detected) |
| **P2603_114 (M)** | Sagittal + bicoronal (partial) + lambdoid left | SCS | Targeted Sanger (*FGFRs, TWIST1*), NGS panel (63 genes) | ***POLR2A***  NM_000937.5 | c.4471C>T, p.(Pro1491Ser) | VUS *(novel)* (het, maternal)  Absent in gnomAD, missense, moderately conserved, moderate physicochemical difference, 3/4 tolerated, inherited from unaffected parent | - | + (only Moon) | - | Neurodevelopmental disorder with hypotonia and variable intellectual and behavioral abnormalities (#618603, AD) |  |
|  |  |  |  | ***FAM20C***  NM_020223.4 | c.1182C>G, p.(Asp394Glu) | VUS (het, carrier, no other variant detected)  0.034757% (30) in gnomAD, missense in protein domain, highly conserved, small physicochemical difference, 3/4 damaging. | - | + (only Moon) | - | Raine syndrome (#259775, AR), Association to CS (Wilkie et al., 2010, Whyte et al., 2017) |  |
| **P2605_168 (M)** | Bicoronal | SCS (Saethre-Chotzen-like) | HaloPlex NGS panel (12 genes) + MLPA, NGS panel (63 genes) | ***PRRX1***  NM_022716.4 | c.283C>T, p.(Arg95*) | VUS *(novel)* (het, maternal)  Absent in gnomAD, truncating in protein domain, inherited from unaffected parent | - | + (only Moon) | - | Agnathia-otocephaly complex (#202650, AD) |  |
|  |  |  |  | ***TAOK1***  NM_020791.4 | c.2410C>G, p.(Gln804Glu) | VUS *(novel)* (het, maternal)  Absent in gnomAD, missense in protein domain, highly conserved, small physicochemical difference, 2/4 damaging, inherited from unaffected parent | - | + (only Moon) | - | Developmental delay with or without intellectual impairment or behavioral abnormalities (#619575, AD) |  |
| **P2605_104 (M)** | Pansynostosis | SCS (Frontonasal dysplasia, Sweeney-Cox-like) | Targeted NGS panel (63 genes) | ***MAP3K7***  NM_145331.2 | c.841G>T, p.(Val281Leu) | VUS *(novel)* (het)  Absent in gnomAD, missense in protein domain, highly conserved, small physicochemical difference, 1/4 damaging | - | + | - | Cardiospondylocarpofacial syndrome (#157800, AD) |  |
|  |  |  |  |  |  |  |  |  |  | Frontometaphyseal dysplasia 2 (#617137, AD) |  |
|  |  |  |  | ***FREM2***  NM_207361.6 | c.9468G>C, p.(Met3156Ile) | VUS (het, carrier, no other variant detected)  0.00079744% (1) in gnomAD, missense, highly conserved, small physicochemical difference, 4/4 tolerated | - | + (only Moon) | - | Fraser syndrome 2 (#617666, AR) |  |
| **P2605_134 (F)** | Unicoronal right + sagittal + lambdoid bilateral | SCS^3^ | Targeted Sanger (FGFRs, TWIST1), NGS panel (63 genes) | ***TET3***  NM_001287491.2 | c.712_713del, p.(Gly238Thrfs*6) | VUS *(novel)* (het, compound?)  Absent in gnomAD, truncating frameshift | - | + (only Moon) | - | Beck-Fahrner syndrome (#618798, AR, AD) |  |
|  |  |  |  |  | c.718del, p.(Arg240Glyfs*28) | VUS *(novel)*  (het, compound?)  Absent in gnomAD, truncating frameshift |  |  |  |  |  |
|  |  |  |  | ***WDR19***  NM_025132.4 | c.2632C>T, p.(Arg878Cys) | VUS (het, carrier, no other variant detected)  0.0049938% (7) in gnomAD, missense, moderately conserved, large physicochemical difference, 3/4 damaging | + | + | - | Cranioectodermal dysplasia 4 (#614378, AR) |  |
| **P_10 (M)** | Unicornal right | SCS^3^ | In silico panel WGS (29 genes) + MLPA | ***TGFBR1***  NM_004612.3 | c.364G>C, p.(Gly122Arg) | VUS *(novel)* (het)  Absent in gnomAD, missense in protein domain, highly conserved, moderate physicochemical difference, 2/4 damaging | + | + | - | Loeys-Dietz syndrome 1(#609192, AD) |  |
|  |  |  |  | ***KMT2C***  NM_170606.3 | c.7040A>G, p.(Gln2347Arg) | VUS (het)  Absent in gnomAD (present in dbSNP), missense, weakly conserved, small physicochemical difference, 4/4 tolerated. | - | + (only Moon) | - | Kleefstra syndrome 2 (#617768, AD) |  |
|  |  |  |  | ***GINS2***  NM_016095.3 | c.130G>A, p.(Val44Met) | VUS (het, carrier, no other variant detected)  0.0008531% (1) in gnomAD, missense in protein domain, highly conserved, small physicochemical difference, 4/4 damaging. | - | + (only Moon) | - | Meier-Gorlin syndrome with craniosynostosis (Nabais Sá et al., 2022) |  |
|  |  |  |  | ***ABCC8***  NM_000352.6 | c.62T>A, p.(Val21Asp) | Likely pathogenic (het)  0.011035% (15) in gnomAD, missense, moderately conserved, large physicochemical difference, 4/4 damaging, reported as pathogenic in ClinVar (VCV000495835.5) and HGMD (CM062384) | - | + (only Moon) | - | Hyperinsulinemic hypoglycemia, familial, 1 (#256450, AD) |  |
| **P_18 (F)** | Right unicoronal | SCS^3^ | In silico panel WES (29 genes) + MLPA | ***ZBTB20***  NM_001164342.1 | c.974G>C, p.(Gly325Ala) | VUS *(novel)* (het)  Absent in gnomAD, missense, highly conserved, small physicochemical difference, 3/4 damaging. | - | + (only Moon) | - | Primrose syndrome (#259050, AD) |  |
|  |  |  |  | ***H4C5***  NM_003545.4 | c.10C>T, p.(Arg4Cys) | VUS *(novel)* (het)  Absent in gnomAD, missense in protein domain, highly conserved, large physicochemical difference, predictions not available | - | + (only Moon) | - | Tessadori-van Haaften neurodevelopmental syndrome 3 (# 619950, AD) |  |
| **P_21 (F)^4^** | Bicoronal | SCS | In silico panel WGS (29 genes) + MLPA | ***GCK***  NM_000162.5 | c.1363G>T, p.(Val455Leu) | Pathogenic (het, not present in mother, father not available)  Absent in gnomAD, missense in protein domain, moderately conserved, small physicochemical difference, 1/4 damaging, reported as pathogenic in HGMD (CM2017407) | - | + | - | Hyperinsulinemic hypoglycemia, familial, 3 (#602485, AD) |  |
| **P2605_186 (M)** | Unicoronal right + lambdoid (partial)(?) | SCS^3^  (Saethre-Chotzen-like) | Targeted Sanger + MLPA of *TWIST1*, NGS panel (63 genes) | ***NSD1***  (observed at targeted NGS, but not reported)  NM_022455.4 | c.-18+2T>G, p.? | VUS *(novel)* (het, paternal)  Absent in gnomAD, predicted to affect splicing of exon 1 (non-coding), inherited from parent with similar phenotype | + | + | - | Sotos syndrome (#117550, AD) |  |
|  |  |  |  | ***FAM20C***  NM_020223.4 | c.389G>A, p.(Arg130Lys) | VUS (het, carrier, no other variant detected)  0.0033174% (2) in gnomAD, missense, weakly conserved, small physicochemical difference, 4/4 tolerated | - | + (only Moon) | - | Raine syndrome (#259775, AR), Association to CS (Wilkie et al., 2010, Whyte et al., 2017) |  |
| **P_15 (F)** | Sagittal + lambdoid bilateral (Mercedes synostosis) | SCS | In silico panel WGS (29 genes) + MLPA | ***NSD1***  NM_022455.4 | c.2952C>A, p.(Asp984Glu) | VUS *(novel)* (het)  Absent in gnomAD, weakly conserved, small physicochemical difference, 4/4 tolerated | + | + | - | Sotos syndrome (#117550, AD) |  |
|  |  |  |  | ***ASH1L***  NM_018489.3 | c.5889A>T, p.(Glu1963Asp) | VUS *(novel)* (het)  Absent in gnomAD, missense, moderately conserved, small physicochemical difference, 2/4 tolerated. | - | + (only Moon) | - | Intellectual developmental disorder, autosomal dominant 52 (#617796, AD) |  |
|  |  |  |  | ***WDR35***  NM_001006657.1 | c.1204G>A, p.(Glu402Lys) | VUS (het, carrier, no other variant detected)  0.0049606% (7) in gnomAD, missense in protein domain, moderately conserved, small physicochemical difference, 1/4 damaging | - | + (only Moon) | - | Cranioectodermal dysplasia 2 (#613610, AR) |  |
| **P_16 (F)** | Unicoronaleft | SCS | In silico panel WGS (29 genes) + MLPA | ***KDM6B***  NM_001080424.1 | c.4909G>A, p.(Val1637Met) | VUS *(novel)* (het)  Absent in gnomAD, missense, weakly conserved, small physicochemical difference, 2/4 tolerated | + | + (only Moon) | - | Neurodevelopmental disorder with coarse facies and mild distal skeletal abnormalities (#618505) |  |
| **P_13 (M)** | Bicoronal + sagittal | SCS | In silico panel WGS (29 genes) + MLPA | ***SPRY1***  NM_001375410.1 | c.530A>C, p.(His177Pro) | VUS (het)  0.023063% (29) in gnomAD, missense, highly conserved, moderate physicochemical difference, 3/4 damaging, assumed inherited from unaffected parent (frequency in gnomAD) | + | - | - | Craniosynostosis, *SPRY1*-related (PMID: 27606499, AD) | *P4HB* (Table S6) |
|  |  |  |  | ***IL11RA***  NM_001142784.2 | c.710G>A, p.(Arg237Gln) | VUS (het, carrier, no other variant detected)  0.012026% (17) in gnomAD, missense in protein domain, weakly conserved, small physicochemical difference, 1/4 damaging, reported as VUS in Clinvar (VCV001305704.1) | + | + | - | Craniosynostosis and dental anomalies (#614188, AR) |  |
|  |  |  |  | ***SOS1***  NM_005633.3 | c.2945G>A, p.(Arg982Gln) | VUS (*novel*) (het, paternal)  Absent in gnomAD, missense in protein domain, highly conserved, small physicochemical difference, 1/4 damaging, reported as VUS in ClinVar (VCV000496301.2), inherited from a priori unaffected parent | - | + (only Moon) | - | Noonan syndrome 4 (#610733, AD) |  |
|  |  |  |  | ***NOTCH2***  NM_024408.3 | c.6941G>C, p.(Ser2314Thr) | VUS *(novel)* (het, paternal)  Absent in gnomAD, missense in protein domain, moderately conserved, small physicochemical difference, 1/4 damaging, inherited from a priori unaffected parent | + | + (only Moon) | - | Alagille syndrome 2 (#610205, AD) |  |

^1^Genotype frequency in control population (gnomAD), effect at protein level, location in protein domain, nucleotide/amino acid evolutionary conservation, physiochemical difference between amino acids, no/4 – no of in silico prediction programs assessing the variant as damaging/tolerated per total no of programs – 4: SIFT, MutationTaster, PolyPhen-2: HumDiv and HumVar^.^

^2^ See Topa et al., 2020 and Topa et al., 2022.

^3^Borderline cases – milder craniofacial changes +/- speech delay or other minor extracranial features which suggested a syndromic form of CS.

^4^WGS Duo with the patient’s mother.

“-“ no detection; “+” variant detected by method; ClinVar – Clinical Genome Resource (database of variants associated with human disease); F – female; gnomAD - The Genome Aggregation Database; het – heterozygous; M – male; MLPA - multiplex ligation-dependent probe amplification; VUS – variant of uncertain significance.
