# Supplementary material for: The value of genome-wide analysis in craniosynostosis
Source: Front Genet. 2024 Jan 22;14:1322462. doi: 10.3389/fgene.2023.1322462 (PMC10839781; doi:10.3389/fgene.2023.1322462)
Supplement: Supplementary file 1 [file DataSheet1.zip › Table S4.DOCX]

Supplementary Table 4

Possibly relevant variants (including modulator effect) observed in patients with NCS and no detected causal variants explaining the CS.

| **Patient no (gender)** | **Sutural pattern** | **Analyses prior to inclusion in the study** (on both clinical and research bases) | **Gene**  (transcript) | **Variant annotation - cDNA, protein level/genomic position for CNVs** | **Variant classification according to ACMG criteria** (novelty, zygosity, inheritance, molecular aspects^1^) | **Detection by screening method** | | | **Associated relevant disorder** (OMIM, PubMed – PMID, Inheritance pattern) | **Previously published variants^2^ confirmed by WES/WGS** |
| --- | --- | --- | --- | --- | --- | --- | --- | --- | --- | --- |
|  |  |  |  |  |  | **In-silico panel on WGS/WES** (133 genes) | **HPO-term analysis with Moon/Alissa software** | **CNV analysis** (Alissa + IGV) |  |  |
| **P_11 (F)^3^** | Unicoronal left | In silico panel WGS (29 genes) + MLPA | ***ERF***  NM_006494.4 | c.993C>G, p.(Phe331Leu) | VUS *(novel)* (het, maternal)  Absent in gnomAD, missense, highly conserved, small physicochemical difference, 3/4 damaging, inherited from a priori unaffected parent. | + | + | - | Craniosynostosis 4 (#600775, AD) |  |
| **P_6 (M)^3,4^** | Sagittal + bicoronal (partial) | In silico panel WES (29 genes) + MLPA | ***CHD7***  NM_017780.3  Present in P_7 and P_8 (brother and mother) | c.3206G>A, p.(Arg1069Gln) | VUS (het, maternal)  0.0014294% (2) in gnomAD, missense in protein domain, highly conserved, small physicochemical difference, 1/4 damaging, reported as VUS in ClinVar (VCV000836902.3) | + | + | - | CHARGE syndrome (#214800, AD) |  |
|  |  |  | ***ALPL***  NM_001177520.2  Present in P_7 and P_8 (brother and mother) | c.935C>A, p.(Thr312Asn) | Likely pathogenic (het, carrier, maternal, no other variant detected)  0.0015906% (2) in gnomAD, missense in protein domain, highly conserved, small physicochemical difference, 2/4 damaging, likely pathogenic in ClinVar (VCV001224321.2) | + | + | - | Hypophosphatasia, infantile (#241500, AR) |  |
| **P_7 (M)^3,4^ –** P_6’s brother  and  **P_8 (F)^3,4^ –** P_6’ and P_7’s mother | Sagittal | In silico panel WGS (29 genes) + MLPA  (mother analyzed only as part of the Quatro WGS) | ***FBN1***  NM_000138.4  Present in P_8 (mother) | c.7412C>T, p.(Pro2471Leu) | VUS (het, maternal)  0.00079568% (1) in gnomAD, missense in protein domain, highly conserved, moderate physicochemical difference, 4/4 damaging, reported as VUS by ClinVar (VCV000654126.3) | + | + | - | Marfan syndrome (#154700, AD) |  |
|  |  |  | ***IFT122***  NM_052985.3  Present in P_7 (son) | c.3385C>T, p.(Arg1129Cys) | VUS (het, carrier, no other variant detected)  0.0057434% (8) in gnomAD, missense, moderately conserved, large physicochemical difference, 1/4 damaging, VUS in ClinVar (VCV000631906.2) | + | + | - | Cranioectodermal dysplasia 1 (#218330, AR) |  |
| **P_12 (M)** | Unicoronal right | In silico panel WGS (29 genes) + MLPA | ***MEGF8***  NM_001271938.2 | c.2341C>T, p.(Arg781Trp) | VUS (het, carrier, no other variant detected)  0.0024338% (3) in gnomAD, missense, moderately conserved, moderate physicochemical difference, 3/4 damaging | + | + | - | Carpenter syndrome 2 (#614976, AR) |  |
|  |  |  | ***CHD5***  NM_015557.2 | c.2048C>T, p.(Thr683Met) | VUS *(novel)* (het, maternal)  Absent in gnomAD, missense in protein domain, highly conserved, moderate physicochemical difference, 3/4 damaging, inherited from unaffected parent | - | + (only Moon) | - | Parenti-Mignot neurodevelopmental syndrome (#619873, AD) |  |
| **P2605_150 (M)** | Unicoronal right + sagittal | Targeted NGS panel (63 genes) | ***SPRY1***  NM_001375410.1 | c.308del, p.(Pro103Glnfs*9) | VUS *(novel)* (het, maternal)  Absent in gnomAD, truncating frameshift | + | - | - | Craniosynostosis, *SPRY1*-related (PMID: 27606499, AD) |  |
|  |  |  | ***P4HB***  NM_000918.3 | c.802T>G, p.(Tyr268Asp) | VUS (het)  0.0091909% (13) in gnomAD, missense in protein domain, moderately conserved, large physicochemical difference, 2/4 damaging, assumed inherited from unaffected parent (frequency in gnomAD) | + | + | - | Cole-Carpenter syndrome 1 (#112240, AD) |  |
|  |  |  | ***RECQL4***  NM_004260.3  (observed at targeted NGS, but not reported) | c.638G>A, p.(Gly213Asp) | VUS (het, carrier, no other variant detected)  0.0063702% (1) in gnomAD, missense, weakly conserved, moderate physicochemical difference, 3/4 tolerated | + | + | - | Baller-Gerold syndrome (#218600, AR) |  |
| **P2605_181 (F)^3^** | Sagittal + lambdoid bilateral (partial) | Targeted NGS panel (63 genes) | ***SPRY1***  NM_001375410.1 | c.152A>G, p.(Asn51Ser) | VUS (het, maternal)  0.0023857% (3) in gnomAD, missense, highly conserved, small physicochemical difference, small physicochemical difference, 3/4 damaging | + | - | - | Craniosynostosis, *SPRY1*-related (PMID: 27606499, AD) |  |
|  |  |  | ***CBL***  NM_005188.4 | c.2222C>T, p.(Ala741Val) | VUS (het)  0.0035384% (5) in gnomAD, missense, highly conserved, small physicochemical difference, 1/4 damaging, VUS in ClinVar (VCV000561785.3) | - | + (only Moon) | - |  |  |
| **P2605_195 (M)^3^** | Unicoronal right | Targeted NGS panel (63 genes) | ***HUWE1***  NM_031407.7 | c.3451C>T, p.(His1151Tyr) | VUS (hemizygous, maternal)  0.0034639% (2) in gnomAD (het), missense, moderately conserved, moderate physicochemical difference, 4/4 not damaging | + | + | - | Intellectual developmental disorder, X-linked, Turner type (#309590) | ***ADAMTSL4*** (het, carrier, no other variant detected) |
| **P2605_150 (M)^3^** | Unicoronal right | HaloPlex NGS panel (12 genes) + MLPA, targeted NGS panel (63 genes) | ***HUWE1***  NM_031407.7 | c.8282C>T, p.(Thr2761Ile) | VUS (hemizygous, maternal)  0.00055023% (1) in gnomAD (hemizygous), missense, weakly conserved, moderate physicochemical difference, 1/4 damaging, observed in a male control | + | - | - | Intellectual developmental disorder, X-linked, Turner type (#309590) |  |
|  |  |  | ***LTBP1***  NM_206943.4 | c.1483G>A, p.(Val495Ile) | VUS (het, carrier, no other variant detected)  0.0084912% (12) in gnomAD, missense, highly conserved, small physicochemical difference, 3/4 damaging. | - | + (only Moon) | - | Cutis laxa, autosomal recessive, type IIE (#619451, AR) |  |
| **P2603_156 (F)** | Sagittal + bicoronal (partial) | Targeted NGS panel (63 genes) | ***TAOK1***  NM_020791.4 | c.2767G>C, p.(Gly923Arg) | VUS *(novel)* (het, not present in mother, father not available)  Absent in gnomAD, missense in protein domain, highly conserved, Moderate physicochemical difference,1/4 damaging | - | + (only Moon) | - | Developmental delay with or without intellectual impairment or behavioral abnormalities (#619575, AD) |  |
| **P2603_129 (F)^3^** | Unicoronal right | Targeted NGS panel (63 genes) | ***PLXNA1***  NM_032242.3 | c.5242C>T, p.(Arg1748Cys) | VUS/Likely pathogenic^3^ (het, reported de novo by other sources)  Absent in gnomAD, missense in protein domain, highly conserved, large physicochemical difference, 4/4 damaging, predicted effect on splicing, reported as likely pathogenic in ClinVar (VCV000867241.1) and HGMD (CM2122787) | - | + (only Moon) | - | Neurodevelopmental disorder, *PLXNA1-*related (PMID: 34054129, AD, AR) |  |
|  |  |  | ***IRX5***  NM_005853.6 | c.277A>G, p.(Met93Val) | VUS (het, carrier, no other variant detected)  0.0028409% (4) in gnomAD, missense, highly conserved, small physicochemical difference | - | + | - | Hamamy syndrome (#611174, AR) |  |
| **P2605_174 (F)** | Unicoronal right | Targeted NGS panel (63 genes) | ***LTBP1***  NM_206943.2 | c.4748C>T, p.(Thr1583Met) | VUS (het, compound?)  0.012023% (17) in gnomAD, missense in protein domain, weakly conserved, moderate physicochemical difference, 2/4 tolerated | - | + (only Moon) | - | Cutis laxa, autosomal recessive, type IIE (#619451, AR) | ***ERF, PTCH1*** |
|  |  |  |  | c.5018G>A, p.(Arg1673Gln) | VUS (het, compound?)  0.011156% (14) in gnomAD, missense in protein domain, highly conserved, small physicochemical difference, 2/4 damaging |  |  |  |  |  |
|  |  |  | ***TAB2***  NM_015093.5 | c.1873C>G, p.(Pro625Ala) | VUS *(novel)* (het)  Absent in gnomAD, missense, highly conserved, small physicochemical difference, 4/4 damaging | - | + (only Moon) | - | Congenital heart defects, nonsyndromic, 2 (# 614980), Frontometaphyseal dysplasia (PMID: 27426733) |  |
| **P2605_113 (F)** | Bicoronal | Targeted NGS panel (63 genes) | ***ADAMTSL4***  (observed at targeted analysis, but not reported)  NM_001288608.1 | c.362G>T, p.(Arg121Met) | VUS *(novel)* (het, carrier, no other variant detected)  Absent in gnomAD, missense in protein domain, highly conserved, moderate physicochemical difference, 1/4 damaging | + | - | - | Ectopia lentis et pupillae (#225200, AR), Craniosynostosis *ADAMTSL4*-related (PMID: 35378950, AR) | ***TGBR1, IL11RA*** (carrier, no other variant detected) |
|  |  |  | ***LTBP1***  NM_206943.4 | c.587A>G, p.(Gln196Arg) | VUS *(novel)* (het, carrier, no other variant detected)  Absent in gnomAD, missense in protein domain, moderately conserved, small physicochemical difference, 2/4 tolerated | - | + (only Moon) | - | Cutis laxa, autosomal recessive, type IIE (#619451, AR) |  |
| **P_19 (F)** | Unicoronal right | In silico panel WES (29 genes) + MLPA | ***IFT122***  NM_052985.4 | c.3832G>A, p.(Gly1278Ser) | VUS *(novel)* (het, carrier, no other variant detected, but no CNV-analysis)  Absent in gnomAD, detected in another unrelated unaffected individual at our lab, weakly conserved, small physicochemical difference, 2/4 tolerated | + | + | - | Cranioectodermal dysplasia 1(#218330, AR) |  |
|  |  |  | ***CEP57***  NM_014679.5 | c.751C>T, p.(Pro251Ser) | VUS (het, carrier, no other variant detected)  0.017024% (24) in gnomAD, missense in protein domain, moderately conserved, moderate physicochemical difference, 4/4 tolerated, VUS in ClinVar (VCV000472260.4) | - | + (only Moon) | - | Mosaic variegated aneuploidy syndrome 2 (#614114, AR) |  |
| **P2603_135 (F)^3^** | Unicoronal left | Targeted NGS panel (63 genes) | ***IFT122***  (observed at targeted analysis, but not reported)  NM_052985.4 | c.1729C>T, p.(Arg577Cys) | VUS (het, carrier, no other variant detected)  0.072548% (102) in gnomAD, missense in protein domain, highly conserved, large physicochemical difference, 1/4 damaging, VUS in ClinVar (VCV000901925.3) | + | + (only Moon) | - | Cranioectodermal dysplasia 1(#218330, AR) |  |
| **P2605_163 (M)^3^** | Unicoronal left | Targeted NGS panel (63 genes) | ***IFT122***  (observed at targeted analysis, but not reported)  NM_052985.4 | c.2552_2553del, p.(Leu851Argfs*4) | VUS (*novel*) (het, carrier, no other variant detected)  Absent in gnomAD, truncating frameshift | + | + | - | Cranioectodermal dysplasia 1(#218330, AR) |  |
|  |  |  | ***OGDHL***  NM_018245.3 | c.791G>A, p.(Arg264Gln) | VUS (het, carrier, no other variant detected)  0.011476% (16) in gnomAD, missense in protein domain, highly conserved, small physicochemical difference, 4/4 damaging | - | + (only Moon) | - | Yoon-Bellen neurodevelopmental syndrome (#619701, AR) |  |
| **P2605_183 (F)** | Unicoronal left | Targeted NGS panel (63 genes) | ***RAB23***  (observed at targeted analysis, but not reported)  NM_016277.5 | c.546A>C, p.(Glu182Asp) | VUS (het, carrier, no other variant detected)  0.012749% (18) in gnomAD, missense in protein domain, highly conserved, small physicochemical difference, 4/4 tolerated | + | + | - | Carpenter syndrome 1 (#201000, AR) |  |
| **P2603_163 (F)** | Unicoronal left + ipsilateral frontosphenoidal | Targeted NGS panel (63 genes) | ***FBN1***  (observed at targeted analysis, but not reported)  NM_000138.4 | c.353A>G, p.(His118Arg) | VUS (het)  0.007182% (9) in gnomAD, missense in protein domain, highly conserved, small physicochemical difference, 2/4 damaging, VUS in ClinVar (VCV000200144.7) | + | + | - | Marfan syndrome (#154700, AD) |  |
|  |  |  | ***IFT140***  NM_014714.4 | c.3830G>A, p.(Arg1277Gln) | VUS (het, carrier, no other variant detected)  0.0071596% (9) in gnomAD, missense, highly conserved, small physicochemical difference, 3/4 tolerated, VUS in ClinVar (VCV000281775.6) | + | + | - | Short-rib thoracic dysplasia 9 with or without polydactyly (#266920, AR) |  |
| **P2605_189 (M)^3^** | Unicoronal right | Targeted NGS panel (63 genes) | ***KMT2D***  (observed at targeted analysis, but not reported)  NM_003482.4 | c.14183G>A, p.(Gly4728Asp) | VUS *(novel)*  Absent in gnomAD, missense, weakly conserved, moderate physicochemical difference, 4/4 tolerated | + | + | - | Kabuki syndrome 1 (#147920, AD) |  |
|  |  |  | ***WDR19***  (observed at targeted analysis, but not reported)  NM_025132.4 | c.3533G>A, p.(Arg1178Gln) | Likely pathogenic (het, carrier, no other variant detected)  0.012693% (17) in gnomAD, missense, moderately conserved, small physicochemical difference, 3/4 damaging, pathogenic/VUS in ClinVar (VCV000127158.10) | + | + | - | Cranioectodermal dysplasia 4 (#614378, AR) |  |
| **P2603_119 (M)** | Unicoronal right + sagittal (partial) | Targeted Sanger (FGFRs, TWIST1, NGS panel (63 genes) | ***CDC45***  NM_001178010.2 | c.539G>A, p.(Arg180Gln) | VUS (het, carrier, no other variant detected)  0.01491% (21) in gnomAD, missense, moderately conserved, small physicochemical difference, 2/4 tolerated | - | + (only Moon) | - | Meier-Gorlin syndrome 7 (#617063, AR) |  |
| **P_22 (M)** | Unicoronal right | In silico panel WGS (29 genes) + MLPA | ***SEC24D***  NM_014822.4 | c.185C>T, p.(Pro62Leu) | VUS (het, carrier, no other variant detected)  0.00081127% (1) in gnomAD, missense, weakly conserved, moderate physicochemical difference, 4/4 tolerated | + | + | - | Cole-Carpenter syndrome 2 (#616294, AR) |  |
|  |  |  | ***SCARF2***  NM_153334.4 | c.871G>A, p.(Gly291Ser) | VUS (het, carrier, no other variant detected)  0.0055915% (6) in gnomAD, missense in protein domain, highly conserved, small physicochemical difference, 1/4 tolerated | - | + | - | Van den Ende-Gupta syndrome (#600920, AR) |  |
|  |  |  | ***OGDHL***  NM_018245.3 | c.2219C>T, p.(Thr740Met) | VUS (het, carrier, no other variant detected)  0.020514% (29) in gnomAD, missense in protein domain, highly conserved, moderate physicochemical difference, 4/4 damaging | - | + (only Moon) | - | Yoon-Bellen neurodevelopmental syndrome (#619701, AR) |  |
| **P_20 (F)^3^** | Unicoronal right | In silico panel WES (29 genes) + MLPA | ***IFT140***  NM_014714.4 | c.2767T>G, p.(Tyr923Asp) | VUS *(novel)* (het, carrier, no other variant detected, but no CNV-analysis)  Absent in gnomAD, missense, moderately conserved, large physicochemical difference, 1/4 damaging, possibly splice effect | + | + | - | Short-rib thoracic dysplasia 9 with or without polydactyly (#266920, AR) |  |
|  |  |  | ***RECQL4***  NM_004260.3 | c.358G>C, p.(Gly120Arg) | VUS (het, carrier, no other variant detected, but no CNV-analysis)  0.01384% (14) in gnomAD, missense, weakly conserved, moderate physicochemical difference, 3/4 tolerated, VUS in ClinVar (VCV000528936.6) | + | + | - | Baller-Gerold syndrome (#218600,AR) |  |
|  |  |  | ***PTPN11***  NM_002834.5 | c.1226G>C, p.(Gly409Ala) | VUS (het)  0.012759% (16) in gnomAD, missense in protein domain, highly conserved, small physicochemical difference, 2/4 damaging, VUS in ClinVar (VCV000044596.8) | + | - | - | Noonan syndrome 1(#163950, AD) |  |
| **P2603_161 (F)** | Unicoronal right | Targeted NGS panel (63 genes) | ***SCARF2***  NM_153334.4 | c.2599G>A, p.(Ala867Thr) | VUS (het, carrier, no other variant detected)  0.0040669% (2) in gnomAD, missense, weakly conserved, small physicochemical difference, 1/4 tolerated | - | + | - | Van den Ende-Gupta syndrome (#600920, AR) |  |
|  |  |  | ***DMP1***  NM_004407.4 | c.55-5T>C  p.(?) | VUS (*novel*) (het, carrier, no other variant detected)  Absent in gnomAD, weakly conserved nucleotide, predicted impact on splicing (low) | - | + (only Alissa) | - | Hypophosphatemic rickets 1 (#241520, AR) |  |
|  |  |  | ***RECQL4***  (observed at targeted analysis, but not reported)  NM_004260.3 | c.1830C>A, p.(Leu610=) | VUS *(novel)* (het, carrier, no other variant detected)  Absent in gnomAD, synonymous in protein domain, nucleotide not conserved, no predicted impact on splicing | + | + | - | Baller-Gerold syndrome (#218600,AR) |  |
|  |  |  | ***FAM20C***  NM_020223.4 | c.764C>T, p.(Ser255Phe) | VUS (het, carrier, no other variant detected)  0.002453% (3) in gnomAD, missense, moderately conserved, large physicochemical difference, 2/4 damaging. | - | + (only Moon) | - | Raine syndrome (#259775, AR), Association to CS (Wilkie et al., 2010, Whyte et al., 2017) |  |
|  |  |  | ***TCF20***  NM_005650.4 | c.3023A>G, p.(Gln1008Arg) | VUS *(novel)* (het)  Absent in gnomAD, missense, moderately conserved, small physicochemical difference, 2/4 damaging | - | + (only Moon) | - | Developmental delay with variable intellectual impairment and behavioral abnormalities (#618430, AD) |  |
| **P2605_187 (F)^3^** | Sagittal | Targeted NGS panel (63 genes) | ***DMP1***  NM_004407.4 | c.428C>T, p.(Thr143Ile) | VUS (het, carrier, no other variant detected)  0.050255% (71) in gnomAD, missense, moderately conserved, moderate physicochemical difference, 4/4 tolerated, VUS in ClinVar (VCV000349973.3) | - | + | - | Hypophosphatemic rickets 1 (#241520, AR) |  |
|  |  |  | ***LTBP4***  NM_001042544.1 | c.1525C>T, p.(Arg509Cys) | VUS (het, carrier, no other variant detected)  0.11895% (154) in gnomAD, missense, moderately conserved, large physicochemical difference, 3/4 damaging, VUS in ClinVar (VCV000329306.7) | - | + (only Moon) | - | Cutis laxa, autosomal recessive, type IC (# 613177, AR) |  |
|  |  |  | ***SP7***  NM_001173467.3  (co-segregates with other variant on same allele according to the genomic data) | c.799C>T, p.(Arg267Cys) | VUS (het, carrier, in *cis* with other variant)  0.036591% (51) in gnomAD, missense, highly conserved, large physicochemical difference, 2/4 damaging, VUS in ClinVar (VCV001302961.2) | - | + (only Moon) | - | Osteogenesis imperfecta, type XII (#613849, AR) |  |
|  |  |  | ***SP7***  NM_001173467.3  (co-segregates with other variant on same allele according to the genomic data) | c.839G>T, p.(Arg280Leu) | VUS (het, carrier, in *cis* with other variant)  0.038033% (53) in gnomAD, missense, highly conserved, moderate physicochemical difference, 4/4 damaging. |  |  |  |  |  |
| **P2603_152 (M)** | Left coronal + sagittal | Targeted NGS panel (63 genes) | ***FAM20C***  NM_020223.4 | c.1654G>A, p.(Val552Ile) | VUS (het, carrier, no other variant detected)  0.018619% (16) in gnomAD, missense in protein domain, weakly conserved, small physicochemical difference, 4/4 tolerated. | - | + (only Moon) | - | Raine syndrome (#259775, AR), Association to CS (Wilkie et al., 2010, Whyte et al., 2017) |  |
| **P2603_153 (F)** | Unicoronal left | Targeted NGS panel (63 genes) | ***MEGF8***  (observed at targeted analysis, but not reported)  NM_001271938.2 | c.4927A>G, p.(Asn1643Asp) | VUS (het, carrier, no other variant detected)  0.0015952% (2) in gnomAD, missense in protein domain, highly conserved, small physicochemical difference, 2/4 damaging | + | + | - | Carpenter syndrome 2 (#614976, AR) |  |
|  |  |  | ***PIGO***  NM_032634.4 | c.339T>G, p.(Ile113Met) | VUS (het, carrier, no other variant detected)  0.0055674% (7) in gnomAD, missense in protein domain, weakly conserved, small physicochemical difference, 4/4 tolerated, VUS in ClinVar (VCV000942025.6) | - | + (only Moon) | - | Hyperphosphatasia with mental retardation syndrome 2 (#614749, AR) |  |

^1^ Genotype frequency in control population (gnomAD), effect at protein level, location in protein domain, nucleotide/amino acid evolutionary conservation, physiochemical difference between amino acids, no/4 – no of in silico prediction programs assessing the variant as damaging/tolerated per total no of programs – 4: SIFT, MutationTaster, PolyPhen-2: HumDiv and HumVar.

^2^See Topa et al., 2020 and Topa et al., 2022

^3^Borderline cases – a syndromic form cannot be excluded due to mild dysmorphic features, lack of or incomplete phenotypic information and/or young age of the patient.

^4^Index-case with mother and brother with similar phenotype included in a WGS Quatro analysis with the unaffected father.

“-“ no detection; “+” variant detected by method; ClinVar – Clinical Genome Resource (database of variants associated with human disease); F – female; gnomAD - The Genome Aggregation Database; het – heterozygous; M – male; MLPA - multiplex ligation-dependent probe amplification; VUS – variant of uncertain significance.
