# Supplementary material for: The value of genome-wide analysis in craniosynostosis
Source: Front Genet. 2024 Jan 22;14:1322462. doi: 10.3389/fgene.2023.1322462 (PMC10839781; doi:10.3389/fgene.2023.1322462)
Supplement: Supplementary file 1 [file DataSheet1.zip › Table S5.DOCX]

Supplementary Table 5

Variants considered unlikely to contribute to the phenotype and/or discarded after segregation analysis.

| **Patient no** (gender) | **Sutural pattern** | **Phenotype** (clinically suspected diagnosis) | **Analyses prior to inclusion in the study** (on both clinical and research basis) | **Gene**  (transcript) | **Variant annotation - cDNA, protein level/genomic position for CNVs** | **Variant classification according to ACMG criteria** (novelty, zygosity, inheritance, molecular aspects^1^) | **Detection by screening method** | | **Associated relevant disorder** (OMIM, PubMed – PMID, Inheritance pattern) |
| --- | --- | --- | --- | --- | --- | --- | --- | --- | --- |
|  |  |  |  |  |  |  | **In-silico panel on WGS/WES** (133 genes) | **HPO-term analysis with Moon/Alissa software** |  |
| **P_9 (M)** | Unicoronal right | SCS | CGH-array, in silico panel WES (29 genes) + MLPA | ***ZNF292***  NM_015021.2 | c.5573C>T, p.(Ser1858Phe) | Likely benign *(novel)* (het, maternal)  Absent in gnomAD, missense, moderately conserved, large physicochemical difference, 1/4 damaging, inherited from unaffected parent | - | + (only Moon) | Intellectual developmental disorder, autosomal dominant 64 (#619188) |
|  |  |  |  | ***POU3F3***  NM_006236.2 | c.863_883dup, p.(Ala288_His294dup) | Likely benign (het, paternal)  Absent in gnomAD (present in dbSNP), in-frame insertion in protein domain, inherited from unaffected parent. | - | + (only Moon) | Snijders Blok-Fisher syndrome (#618604, AD) |
|  |  |  |  | ***CHD8***  NM_001170629.2 | c.6823G>A, p.(Val2275Ile) | Likely benign *(novel)* (het, maternal)  Absent in gnomAD, missense, moderately conserved, small physicochemical difference, 1/4 tolerated, inherited from unaffected parent. | - | +(only Moon) | Intellectual developmental disorder with autism and macrocephaly (#615032, AD) |
| **P2605_102 (M)** | Bicoronal+metopic+sagittal (?) | SCS (Shprintzen-Goldberg-like, atypical) | Targeted NGS panel (63 genes) | ***BPTF***  NM_182641.3 | c.7913A>G, p.(Gln2638Arg) | Likely benign *(novel)* (het)  Absent in gnomAD, missense in protein domain, moderately conserved, small physiochemical difference, 1/4 damaging, alternative molecular basis for disease (Table 1), assumed inherited from unaffected parent | - | + | Neurodevelopmental disorder with dysmorphic facies and distal limb anomalies (#617755, AD) |
| **P2605_132 (F)** | Unicoronal right | SCS (BOS – like) | Targeted Sanger (*FGFR1, FGFR2, FGFR3, TWIST1)* and NGS panel (63 genes) | ***FOXP4***  NM_001012426.1 | c.371_391del, p.(Pro124_Leu130del) | Likely benign (het, paternal)  0.00082664%(1) in gnomAD, in-frame deletion, no predicted impact on splicing, alternative molecular basis for disease (Table 1), inherited from unaffected parent. | - | + (only Moon) | Developmental disorder with speech/language delays and multiple congenital abnormalities (PMID: 33110267, AD) |
| **P_4 (M)** | Bicoronal | SCS | CGH-array, Fragile X, in silico panel WGS(29 genes)+MLPA | ***SHANK3***  NM_033517.1 | c.2809C>T, p.(Arg937Trp) | Likely benign *(novel)* (het)  Absent in gnomAD, missense, moderately conserved, moderate physicochemical difference,1/4 damaging, reported as VUS in ClinVar (VCV001306349.1), alternative molecular basis for the disease (Table 1), assumed inherited from unaffected parent | - | + (only Moon) | Phelan-McDermid syndrome (#606232, AD) |
| **P_5 (F)** | Sagittal | SCS | In silico panel WGS(29 genes)+MLPA | ***ANKRD11***  NM_013275.5 | c.6736C>T, p.(Pro2246Ser) | Likely benign *(novel)* (het, maternal)  Absent in gnomAD, missense, weakly conserved, moderate physicochemical difference, 4/4 tolerated, alternative molecular basis for disease, inherited from unaffected parent. | - | + (only Moon) | KBG syndrome (#148050, AD) |
| **P_13 (M)** | Bicoronal + sagittal | SCS | In silico panel WGS (29 genes) + MLPA | ***P4HB***  NM_000918.3 | c.52G>A, p.(Asp18Asn) | Likely benign (het, paternal)  0.0031443% (1) in gnomAD, missense, weakly conserved, small physicochemical difference, 4/4 tolerated, inherited from unaffected parent (verified by extern laboratory, several individuals heterozygous and even homozygous in their internal database). | + | + | Cole-Carpenter syndrome 1 (#112240, AD) |
| **P2605_136 (M)** | Metopic + sagittal | SCS (Carpenter-like) | Karyotype + FISH 22q11, targeted NGS panel (63 genes) | ***SETD1B***  NM_001353345.2 | c.3758C>A, p.(Pro1253His) | Likely benign *(novel)* (het, paternal)  Absent in gnomAD, missense, moderately conserved, moderate physicochemical difference, 2/4 tolerated, inherited from unaffected parent. | - | + (only Moon) | Intellectual developmental disorder with seizures and language delay (#619000, AD) |

^1^ Genotype frequency in control population (gnomAD), effect at protein level, location in protein domain, nucleotide/amino acid evolutionary conservation, physiochemical difference between amino acids, no/4 – no of in silico prediction programs assessing the variant as damaging/tolerated per total no of programs – 4: SIFT, MutationTaster, PolyPhen-2: HumDiv and HumVar.

“-“ no detection; “+” variant detected by method; ClinVar – Clinical Genome Resource (database of variants associated with human disease); F – female; gnomAD - The Genome Aggregation Database; het – heterozygous; M – male; MLPA - multiplex ligation-dependent probe amplification; VUS – variant of uncertain significance.
